# CP-25 inhibits the hyperactivation of rheumatic synoviocytes by suppressing the switch in G_αs_-G_αi_ coupling to the β_2_-adrenergic receptor

**DOI:** 10.1186/s12964-023-01358-z

**Published:** 2023-11-30

**Authors:** Mingli Ge, Li Wu, Feng He, Yu Tai, Ruhong Fang, Dafei Han, Paipai Guo, Hao Liu, Yong Hu, Shenglin Xu, Wei Wei, Qingtong Wang

**Affiliations:** 1 Institute of Clinical Pharmacology, Anhui Medical University, Key Laboratory of Anti-Inflammatory and Immune Medicine, Ministry of Education, Collaborative Innovation Center of Anti-Inflammatory and Immune Medicine, Hefei, 230032 China; 2 Hefei Cancer Hospital, Chinese Academy of Sciences, Hefei, 230031 China; 3 School of Pharmacy, Bengbu Medical College, Bengbu, 233030 China; 4 Department of Orthopaedics, The First Affiliated Hospital of Anhui Medical University, Hefei, 230032 China

**Keywords:** β_2_ adrenergic receptor, G_αs_-G_αi_ coupling switch, Paeoniflorin-6′-O-benzene sulfonate, Fibroblast-like synoviocytes, Rheumatoid arthritis

## Abstract

In essence, the β_2_ adrenergic receptor (β_2_AR) plays an antiproliferative role by increasing the intracellular cyclic 3’,5’-adenosine monophosphate (cAMP) concentration through G_αs_ coupling, but interestingly, β_2_AR antagonists are able to effectively inhibit fibroblast-like synoviocytes (FLSs) proliferation, thus ameliorating experimental RA, indicating that the β_2_AR signalling pathway is impaired in RA FLSs via unknown mechanisms. The local epinephrine (Epi) level was found to be much higher in inflammatory joints than in normal joints, and high-level stimulation with Epi or isoproterenol (ISO) directly promoted FLSs proliferation and migration due to impaired β_2_AR signalling and cAMP production. By applying inhibitor of receptor internalization, and small interfering RNA (siRNA) of G_αs_ and G_αi_, and by using fluorescence resonance energy transfer and coimmunoprecipitation assays, a switch in G_αs_-G_αi_ coupling to β_2_AR was observed in inflammatory FLSs as well as in FLSs with chronic ISO stimulation. This G_αi_ coupling was then revealed to be initiated by G protein coupled receptor kinase 2 (GRK2) but not β-arrestin2 or protein kinase A-mediated phosphorylation of β_2_AR. Inhibiting the activity of GRK2 with the novel GRK2 inhibitor paeoniflorin-6′-O-benzene sulfonate (CP-25), a derivative of paeoniflorin, or the accepted GRK2 inhibitor paroxetine effectively reversed the switch in G_αs_-G_αi_ coupling to β_2_AR during inflammation and restored the intracellular cAMP level in ISO-stimulated FLSs. As expected, CP-25 significantly inhibited the hyperplasia of FLSs in a collagen-induced arthritis (CIA) model (CIA FLSs) and normal FLSs stimulated with ISO and finally ameliorated CIA in rats. Together, our findings revealed the pathological changes in β_2_AR signalling in CIA FLSs, determined the underlying mechanisms and identified the pharmacological target of the GRK2 inhibitor CP-25 in treating CIA.

Video Abstract

**Supplementary Information:**

The online version contains supplementary material available at 10.1186/s12964-023-01358-z.

## Background

Rheumatoid arthritis (RA) is the most common type of chronic systemic inflammatory arthritis, affecting 0.5–1% of the global population, with typical clinical manifestations of chronic pain, stiffness and swelling in joints [1]. More than 50% of RA patients become disabled within 10 years after diagnosis due to the significant increases in comorbidity and mortality, and the pathogenesis is still unclear [2]. Synovial tissue is the target of inflammation, and the proliferation and migration of fibroblast-like synoviocytes (FLSs) induced by inflammatory stimulation leads to the further expression of proinflammatory cytokines such as tumour necrosis factor-α (TNF-α), interleukin-1β (IL-1β), and matrix metalloproteinases (MMPs), in turn resulting in destruction of articular cartilage and bone; therefore, the abnormal activation of FLSs, manifested as increased cell viability, migration capacity and invasion capacity, is a critical driver of the progression of RA [3].

The level of intracellular cyclic 3′,5′-adenosine monophosphate (cAMP) is a pivotal controller of cell proliferation and migration. In essence, cAMP activates downstream protein kinase A (PKA) to promote the expression of cell cycle inhibitory proteins while reducing the activity of extracellular regulated protein kinase (ERK) and the expression of cyclin D1/D3, which initiate cell proliferation, leading to the maintenance of FLSs in a resting state. However, under specific conditions, cAMP promotes cell growth by activating ERK [4]. Accumulating evidence has shown that multiple G_αs_-coupled G protein-coupled receptors (GPCRs), including adrenergic receptors (ARs), adenosine receptors, and prostaglandin E_2_ receptors, are expressed on FLSs. However, in the process of RA, the cAMP level in FLSs is substantially reduced, and this decrease is decoupled from the increases in the levels of GPCR ligands, leading to hyperactivation of FLSs. G protein coupled receptor kinase 2 (GRK2)- and β-arrestin2 (βarr2)-induced desensitization and endocytosis of GPCRs on FLSs may contribute to the downregulation of cAMP production in inflammatory FLSs, and further pathomechanisms need to be determined.

Both central and peripheral immune organs are precisely innervated by sympathetic nerves [5]. Activated sympathetic nerves secrete large amounts of epinephrine (Epi) and norepinephrine, which activate ARs in immune cells and regulate the immune response [6]. ARs include α_1_AR, α_2_AR, β_1_AR, β_2_AR and β_3_AR. ARs participate in the pathological process of RA by regulating the activation of T and B lymphocytes and other immune cells [7–9]. In addition to regulating the activation of immune cells, Epi also have an important activating effect on FLSs during inflammation. Studies have shown that plasma levels of Epi and norepinephrine in RA patients are significantly higher than those in healthy people. Treatment of rats with adjuvant arthritis (AA) with the nonselective α-AR blocker phenoxybenzamine, the selective α_1_AR antagonist prazosin, the selective α_2_AR antagonist yohimbine, the nonselective β-AR blocker propranolol, the selective β_1_AR antagonist metoprolol, or the selective β_2_AR antagonists butoxamine and ICI 118551 (ICI) respectively, showed that only the two β_2_AR antagonists effectively reduced arthritis manifestations [10, 11]. These results suggest that the β_2_AR signalling pathway is the key player in inducing the pathological effect of adrenergic stimulation in RA.

It is difficult to understand how G_αs_ couples to β_2_AR, which results in the abundant production of cAMP when β_2_AR activation contributes to inflammation and FLS hyperplasia. Some data have indicated that high-level stimulation with Epi triggers β_2_AR desensitization and internalization via GRK2 and βarr2, leading to suppressed production of cAMP. Our preliminary data revealed that high-level stimulation with isoproterenol (ISO) (1 μM) significantly decreased the intracellular cAMP concentration in rat FLSs; however, blocking ISO-induced β_2_AR internalization using barbadin (Bar) to selectively inhibit the βarr2/β_2_-adaptin interaction only partially restored the production of cAMP [12]. Instead, inhibiting GRK2 activity with either the recognized inhibitor paroxetine (PAR) or a novel inhibitor developed by our group, paeoniflorin-6′-O-benzene sulfonate (CP-25), almost completely restored cAMP production, indicating an additional pathomechanism of GRK2 beyond the regulation of receptor endocytosis [13]. Subsequently, we found that G_αi_ knockdown substantially restored the terbutaline (Ter) response in ISO-treated rat FLSs. Therefore, in the present work, we demonstrated that GRK2-mediated G_αi_ coupling to β_2_AR in inflammatory FLSs exacerbates cAMP signalling inhibition and increases FLS proliferation in the setting of arthritis. Our work provides a comprehensive understanding of the pathological function of GRK2 in arthritis and confirms that selective GRK2 inhibitors, such as CP-25, are promising and effective antirheumatic drugs.

## Materials and methods

### Induction and treatment of collagen-induced arthritis (CIA) in rats

The animal study was approved by the Animal Ethics Committee of the Institute of Clinical Pharmacology, Anhui Medical University. Six- to eight-week-old male Wistar rats (purchased from Shanghai SLAC Laboratory Animal Co., Ltd, Shanghai, China) were housed in a pathogen-free laboratory at the Institute of Clinical Pharmacology, Anhui Medical University. An emulsion of chicken type II collagen (Catalogue #20011, Chondrex, Woodinville, WA, dissolved in 0.1 mol/L acetic acid) and Complete Freund’s adjuvant (CFA; 4 mg/ml, Catalogue #7001, Chondrex, Woodinville, WA) was applied for intradermal injection of rats on Day 0 and Day 7 to establish the rat CIA model. Six normal rats and 6 CIA rats were compared. In the treatment study, when the joints exhibited swelling on Day 14, 15 CIA rats were randomly divided into 3 groups based on the arthritis index through a stratified random sampling method and then subjected to vehicle (Veh; 0.25% sodium carboxymethyl cellulose, CMC-Na; CIA-Veh), CP-25 (C_29_H_32_O_13_S, MW: 620, 50 mg/kg/d, synthesized by Chemistry Lab of Institute of Clinical Pharmacology, Anhui Medical University, Anhui, China; CIA-CP-25), or methotrexate (MTX; 2 mg/kg/3d, MAOXIANG Pharm, Co., Ltd. Changchun, China; CIA-MTX) treatment for 21 days. Five noninjected rats served as normal controls. The body weight and clinical parameters, including the swollen joint count, arthritis index, volume of paw swelling, and global assessment, were evaluated and recorded every 3 days.

### Histopathological examination of joints

After treatment, all rats were sacrificed, and the ankle joints were collected and fixed with formalin for 24 h prior to decalcification in 10% ethylenediaminetetraacetic acid. Four-micron slices of paraffin-embedded joints were stained with H&E, imaged with a 3D HISTECH panoramic scanner and analysed with CaseViewer software 2.4.0.119028 (3DHISTECH Ltd, Budapest, Hungary). Two independent observers evaluated the histological changes in joints, namely, synovial hyperplasia, bone erosion, pannus formation, cell infiltration and cartilage destruction. The pathological score ranged from 0 (no change) to 4 (severe change) based on the scoring standards described previously [14].

### Epi measurement in joints

The Epi concentration in joint homogenates of CIA rats was measured using an enzyme-linked immunosorbent assay (ELISA) kit (Catalogue # CSB-E08678r, CUSABIO, Wuhan, China) according to the operation manual. The absorbance was measured at 450 nm using a Bio Tek ELx808 microplate reader (Lonza Group, Ltd, Basel, Switzerland).

### Primary FLS culture and transfection

Rats were sacrificed and sterilized in 75% alcohol, and synovial tissues from the bilateral knees were collected under sterile conditions. After rinsing in 75% alcohol for 5 min and in PBS three times, the synovial tissues were cut into approximately 1 mm^3^ blocks and attached to the bottom of a culture flask in a cell culture hood. The flask was inverted for 4 h and was then turned upright for continuous culture. After FLSs were spread around the tissue blocks, the tissue blocks were removed, and the FLSs were detached by trypsin. Three to five generations of FLSs were used for the following experiments. For the indicated study, 0.1 μg of β_2_AR short hairpin RNA (shRNA) was added to 5 μl of Opti-MEM (Catalogue # 31985062, Thermo Fisher Scientific, Inc., Waltham, MA, USA), and 0.3 μl of Lipofectamine 2000 transfection reagent (Catalogue # 11668027, Thermo Fisher Scientific, Inc., Waltham, MA, USA) was added to 5 μl of Opti-MEM. The 2 solutions were mixed gently and incubated at room temperature for 2 min before being added to the cells. The small interfering RNA (siRNA) (50 nM) against βarr2, G_αs_, and G_αs_ and the control siRNA (Sangon Co., Ltd, Shanghai, China) were mixed separately with PEI (Mirus, Madison, WI, USA) according to the manufacturer’s instructions and transfected into the cells for 24 h at 37 °C. Green fluorescence could be observed after 48 h of incubation, indicating the successful transfection of β_2_AR shRNA, βarr2 siRNA, G_αs_ siRNA and G_αi_ siRNA. The sequences of specific siRNA were listed in Table 1.

**Table 1:** The sequences of siRNA for βarr2, G_αs_ and G_αi_

Genes	Sense (5'-3')	Antisense (5'-3')
βarr2	5'-GACCGACUGCUGAAGAAGUTT-3'	5'-ACUUCUUCAGCAGUCGGUCTT-3'
G_αs_	5'-CCUACAUGUUAAUGGGUUUTT-3'	5'-AAAGAUUCCAGAGGUCAGGTT-3'
G_αi_	5'-GCUGCAGAGGAAGGCUUUATT-3'	5'-UAAAGCCUUCCUCUGCAGCTT-3'
Control	5'-UUCUCCGAACGUGUCACGUTT-3'	5'-AGGUGACACGUUCGGAGAATT-3'

### FLS viability assay

A cell counting kit-8 (CCK-8) assay was used to evaluate the viability of FLSs. Briefly, FLSs were seeded in a 96-well plate at 5 × 10^4^ cells/well and cultured for 48 h under the indicated treatment conditions. Ten microlitres of CCK8 reagent (Catalogue # BS350A, Biosharp, Guangzhou, China) was added to each well 4 h before the end of the culture period, and the absorbance was measured at 450 nm on a Bio Tek ELX808 microplate reader (Lonza Group, Ltd, Basel, Switzerland).

### Cell migration and invasion assays

Transwell plates were used to evaluate the migration and invasion of FLSs. A total of 5 × 10^4^ FLSs in serum-free Dulbecco’s modified Eagle’s medium (DMEM) were seeded in the upper chamber of a transwell plate, and 500 μl of 10% serum DMEM was added to the bottom chamber. The cells were treated and cultured for 48 h, and the membrane in the upper chamber was washed with phosphate-buffered saline. The cells remaining in the upper chamber were removed by wiping, while the migrated FLSs were fixed with crystal violet solution and counted after photographing. The FLS invasion ability was measured using the same method but with a Matrigel coating on the membrane (Catalogue # 354234, Corning, NY, USA) in the upper chamber of the transwell plate.

### Coimmunoprecipitation (Co-IP)

The interaction of β_2_AR with G_αs_ or G_αi_ was confirmed by co-IP as previously reported [15]. Normal or CIA FLSs or normal FLSs treated with ISO in the presence or absence of the GRK2 inhibitor CP-25 were lysed in NP40 immunoprecipitation buffer supplemented with protease inhibitor cocktails. The cell lysate supernatant was collected after centrifugation at 15,000 × g for 15 min at 4 °C, and the protein concentration was determined by a BCA protein assay kit (Catalogue #23225, Thermo Fisher Scientific Inc., Waltham, MA, USA). One milligram of protein was preincubated with 10 μl of Protein A/G PLUS-Agarose beads (Catalogue # sc-2003, Santa Cruz, CA, USA) and with 2 μg of mouse IgG as the control antibody for 1 h at 4 °C, and the precipitates were then collected by centrifugation at 1000 × g for 1 min at 4 °C. A portion of the supernatant was retained for input analysis. The precleared protein was then incubated with 10 μl of Protein A/G PLUS-Agarose beads preincubated with the anti-β_2_AR antibody (Catalogue # sc-570, Santa Cruz, CA, USA) overnight at 4 °C with rotation. The beads were then precipitated by centrifugation and boiled with 2 × sodium dodecyl sulfate (SDS) loading buffer, and G_αs_, G_αi_ and β_2_AR were detected using Western blotting.

### Western blotting

Proteins from lysed FLSs were collected as mentioned before, separated on a 10% SDS polyacrylamide gel and then transferred to a polyvinylidene fluoride membrane (Millipore Corporation, Billerica, MA). The membrane was blocked in Tris-buffered saline containing 0.05% Tween 20 (TBST) and 5% nonfat milk at 37 °C for 2 h, followed by incubation with a primary antibody against β_1_AR (1:1000, Catalogue # PA1-049, Thermo Fisher Scientific, Waltham, MA, USA), β_2_AR (1:600, Catalogue # sc-570, Santa Cruz Biotechnology, CA, USA), β_3_AR (1:500, Catalogue # YT0363, Immunoway, TX, USA), G_αs_ (1:500, Catalogue # sc-823, Santa Cruz Biotechnology, CA, USA), G_αi_ (1:500, Catalogue # sc-391, Santa Cruz Biotechnology, CA, USA), or GAPDH (1:5000, Catalogue # AF0911, Affinity Biosciences, Changzhou, China) overnight at 4 °C. After washing with TBST. The membrane was incubated with goat anti-rabbit IgG (H + L) horseradish peroxidase (HRP)-linked (1:10,000, Catalogue # S0001, Affinity Biosciences, Changzhou, China) or goat anti-mouse IgG (H + L) HRP-linked (1:10,000, Catalogue # S0002, Affinity Biosciences, Changzhou, China) at 37 °C for 2 h. To evaluate the cellular distribution of β_2_AR, membrane and cytosolic proteins were extracted with a membrane and cytosolic protein extraction kit (Beyotime Biotechnology, Shanghai, China). Enhanced Chemiluminescence Western Blotting Substrate (Catalogue # 32106, Thermo Fisher Scientific, Waltham, MA, USA) was applied for band detection on an ImageQuant LAS 500 Imager (GE Healthcare Systems, Chicago, IL, USA). Protein expression was semiquantified with ImageJ (version 1.42q, NIH) and was normalized to GAPDH expression.

### Quantitative real-time PCR (qRT‒PCR)

Total RNA was extracted from FLSs using TRIzol reagent following the manufacturer’s protocol. Complementary DNA (cDNA) was then synthesized with a cDNA synthesis kit (Catalogue #: 634926, Takara Bio Inc., Otsu, Shiga, Japan), and the specific genes were then amplified from the cDNA templates in a 7500 Real-Time PCR System (Applied Biosystems, Foster City, CA, USA) with Fast SYBR Green Master Mix (Catalogue #: 4385612, Thermo Fisher Scientific, Waltham, MA, USA). The specific primers used for amplification of β_1_AR, β_2_AR, and β_3_AR were listed in Table 2. Changes in expression were calculated by normalization to the corresponding ACTIN levels with the 2^−ΔΔCt^ method.

**Table 2:** Primers for β_1_AR, β_2_AR, and β_3_AR mRNA amplification

Gene	Forward Sequence	Reverse Sequence
β_1_AR	5’-GATCTGGTCATGGGACTGCT-3’	5’-CACGTCTACCGAAGTCCAGA-3’
β_2_AR	5’-CATAACCTCCTTGGCGTGTG-3’	5’-TCGCACCAGAAATTGCCAAA-3’
β_3_AR	5’-GCAGTAGTCCTGTGGAT-3’	5’-GGGCATATTGGAGGCAAAGG-3’
ACTIN	5’-TACAACCTCCTTGCAGCTCC-3’	5’-GGATCTTCATGAGGTAGTCAGTC-3’

### Immunofluorescence staining

Immunofluorescence staining was performed to detect the in situ expression and distribution of the indicated proteins as previously described [16]. FLSs were plated on coverslips and treated for 48 h before fixation with 4% paraformaldehyde for 30 min and permeabilization with 0.5% Triton for 15 min. Subsequently, the cells were blocked with 1% BSA for 30 min and incubated with primary antibodies, including anti-β_1_AR (1:600, Catalogue # sc-568, Santa Cruz Biotechnology, CA, USA), anti-β_2_AR (1:600, Catalogue # sc-570, Santa Cruz Biotechnology, CA, USA), anti-G_αs_ (1:500, Catalogue # sc-823, Santa Cruz Biotechnology, CA, USA), and anti-G_αi_ (1:500, Catalogue # sc-391, Santa Cruz Biotechnology, CA, USA) antibodies, overnight at 4 °C prior to 3 rinses and incubation with goat-anti-mouse Alexa Fluor 488 (1:200, Catalogue # 615–545-214, Jackson ImmunoResearch Inc., West Grove, PA, USA) or goat-anti-rabbit Alexa Fluor 555 (1:200, Catalogue # A-21428, Thermo Fisher Scientific Inc., Waltham, MA, USA) secondary antibodies for 1 h. Finally, coverslips were mounted with a mounting solution containing 4′,6-diamidino-2-phenylindole and then observed on a Leica TCS SP80 confocal microscope (Leica Microsystems, Wetzlar, Germany). β_2_AR coupling with G_αs_ and G_αi_ was semiquantified using the built-in colocalization analysis software module.

### Fluorescence Resonance Energy Transfer (FRET)

A FRET assay was performed to detect intracellular cAMP production upon β_2_AR activation [17]. FLSs were seeded on coverslips in 24-well plates and transfected with 0.5 μg of regular pcDNA-Epac 3 (Reg-ICUE3) plasmid for 36 h. The cells were treated with ISO (final concentration of 1 μM), with or without CGP20712A (CGP, β_1_AR antagonist, 1 μM), ICI (β_2_AR antagonist, 1 μM), SR59230A (SR, β_3_AR antagonist, 100 nM), Bar (10 μM), a PKA inhibitor (PKI, 1 μM), CP-25 (1 μM), or PAR (1 μM) overnight before the end of the transfection period and stimulated acutely with ISO (100 nM), dobutamine (Dob;, β_1_AR agonist, 10 μM) or Ter (β_2_AR agonist, 10 μM). The fluorescence signals in both the 480 nm and 535 nm channels were recorded on a Leica TCS SP80 confocal microscope, and the intensity ratio of cyan fluorescent protein (CFP) to yellow fluorescent protein (YFP) was calculated at different time points. When the level of intracellular cAMP is increased, the CFP/YFP ratio is decreased.

### Statistical analysis

Data were collected from three to five animals per group and were analysed with GraphPad Prism software (version 9, GraphPad Software, Inc., San Diego, CA, USA) and expressed as the means ± standard deviations (SDs). One-way analysis of variance (ANOVA) was used to determine the significance of differences among three or more groups. Two-way ANOVA was used to determine the significance of differences among three or more groups when time was also considered as a variable. Independent* t* tests were used for comparisons between 2 groups. *p* < 0.05 was considered to indicate a significant difference.

## Results

### The elevated Epi level in CIA promotes FLS hyperactivation, accompanied by a reduced βAR response

**Fig. 1:**
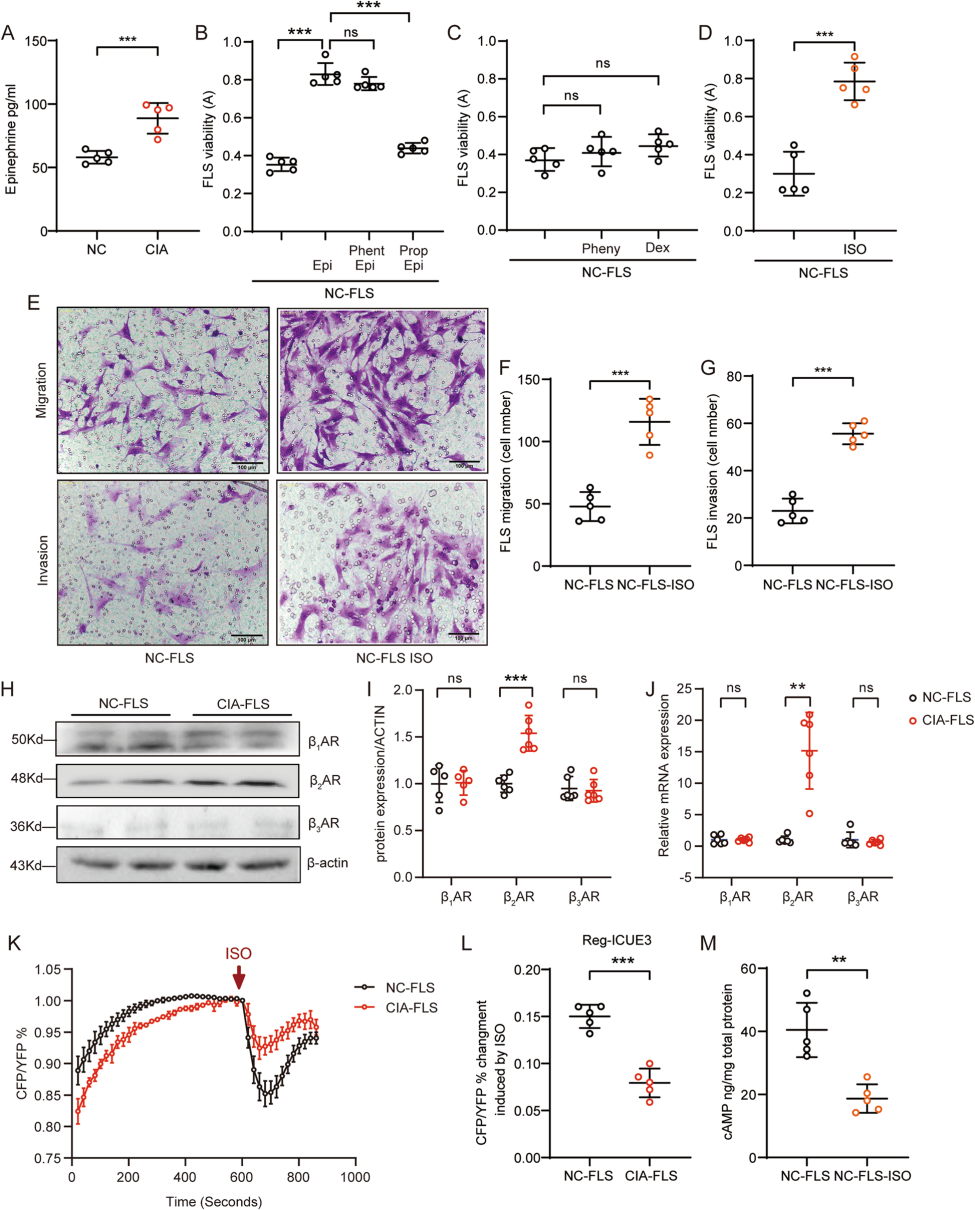
The elevated Epi level in CIA promotes FLS hyperactivation in conjunction with a reduced βAR response. **A** The level of Epi in serum was measured by ELISA. **B** The effect of Phent or Prop pretreatment on Epi-induced FLS viability was measured using a CCK-8 assay. **C** The effect of Pheny or Dex stimulation on FLS viability was detected. **D** The effect of ISO stimulation on FLS viability was detected. **E** The effects of ISO (1 μM) stimulation on FLS migration and invasion were detected by a Transwell assay. Scale bar, 100 μm. **F** Quantification of the number of migrated cells. **G** Quantification of the number of invaded cells. **H** β_1_AR, β_2_AR and β_3_AR expression in FLSs from normal or CIA rats was measured by Western blotting. **I** Analysis of the indicated protein levels. **J** The mRNA levels of β_1_AR, β_2_AR and β_3_AR in both normal and CIA FLSs were measured by qRT‒PCR. **K**, **L** Intracellular cAMP production in living FLSs upon ISO (100 nM) stimulation was monitored in the FRET system, and the CFP/YFP ratio was compared. **M** The intracellular cAMP concentration in rat FLSs treated with Veh or ISO (1 μM) for 48 h was measured by applying a cAMP detection kit. The data are presented as the means ± SEMs; **p < 0.01, ***p < 0.001; *n* = 5–6 animals per group

We previously reported that the serum Epi level is increased in both RA patients and RA animal models [18]. Compared with normal rats, CIA rats exhibited more epinephrine in the joints, and these rats had definite body weight loss; increased global assessment scores, arthritis indexes, and numbers of swollen joints; and paw swelling (Supplementary Fig. 1A-E and Fig. 1A). These findings suggested that adrenergic stress is present at the local site of inflammation. To clarify the impact of high levels of Epi on FLSs and the function of αARs and βARs, we pretreated FLSs with αAR or βAR antagonists prior to high-level stimulation with Epi. The data demonstrated that chronic stimulation with 10 μM Epi markedly promoted the proliferation of normal rat FLSs and that pretreatment with the nonselective α_1_AR and α_2_AR blocker, phentolamine (Phent, 10 μM), was not able to successfully affect Epi-induced FLS proliferation. In contrast, the proliferation of FLSs pretreated with propranolol (Prop) could not be obviously activated in response to Epi stimulation (Fig. 1B). Furthermore, direct activation of α_1_AR with phenylephrine (Pheny, 50 μM) or stimulation of α_2_AR with dexmedetomidine (Dex, 10 μM) for 48 h failed to promote FLS proliferation (Fig. 1C), while treatment with the nonselective βAR agonist ISO (1 μM) induced the activation of FLSs in vitro (Fig. 1D). Chronic ISO stimulation also induced clear migration and invasion of FLSs (Fig. 1E-G). All three subtypes of βARs, β_1_AR, β_2_AR, and β_3_AR, were observed in FLSs; however, β_1_AR and β_3_AR protein expression was not obviously changed but β_2_AR expression was significantly increased in CIA FLSs relative to normal FLSs. Of note, β_3_AR expression in FLSs was quite limited (Fig. 1H and I). Moreover, the mRNA expression levels of the three isotypes were measured by qRT‒PCR. As expected, β_1_AR and β_3_AR mRNA expression was not markedly changed in CIA-FLSs compared with normal FLSs. However, β_2_AR mRNA expression was extremely high in CIA FLSs (Fig. 1J), consistent with the protein level. To test the function of βARs in FLSs during inflammation, 100 nM ISO was used to acutely stimulate either normal FLSs or CIA FLSs, and intracellular cAMP production was monitored in real time in living cells with a FRET system. Interestingly, we found that although β_2_AR expression was upregulated in CIA FLSs, ISO-induced cAMP production was markedly impaired (Fig. 1K and L). Moreover, the cAMP concentration in normal rat FLSs treated with 1 μM ISO for 48 h was much lower than that in Veh-treated normal FLSs, as determined with a cAMP detection kit (Fig. 1M). These data reveal that the βAR response was attenuated in CIA FLSs and that this pathological change may be induced by the high level of Epi in the joint environment.

### Chronic Epi stimulation inhibits cAMP production and activates FLSs through β_2_AR

**Fig. 2:**
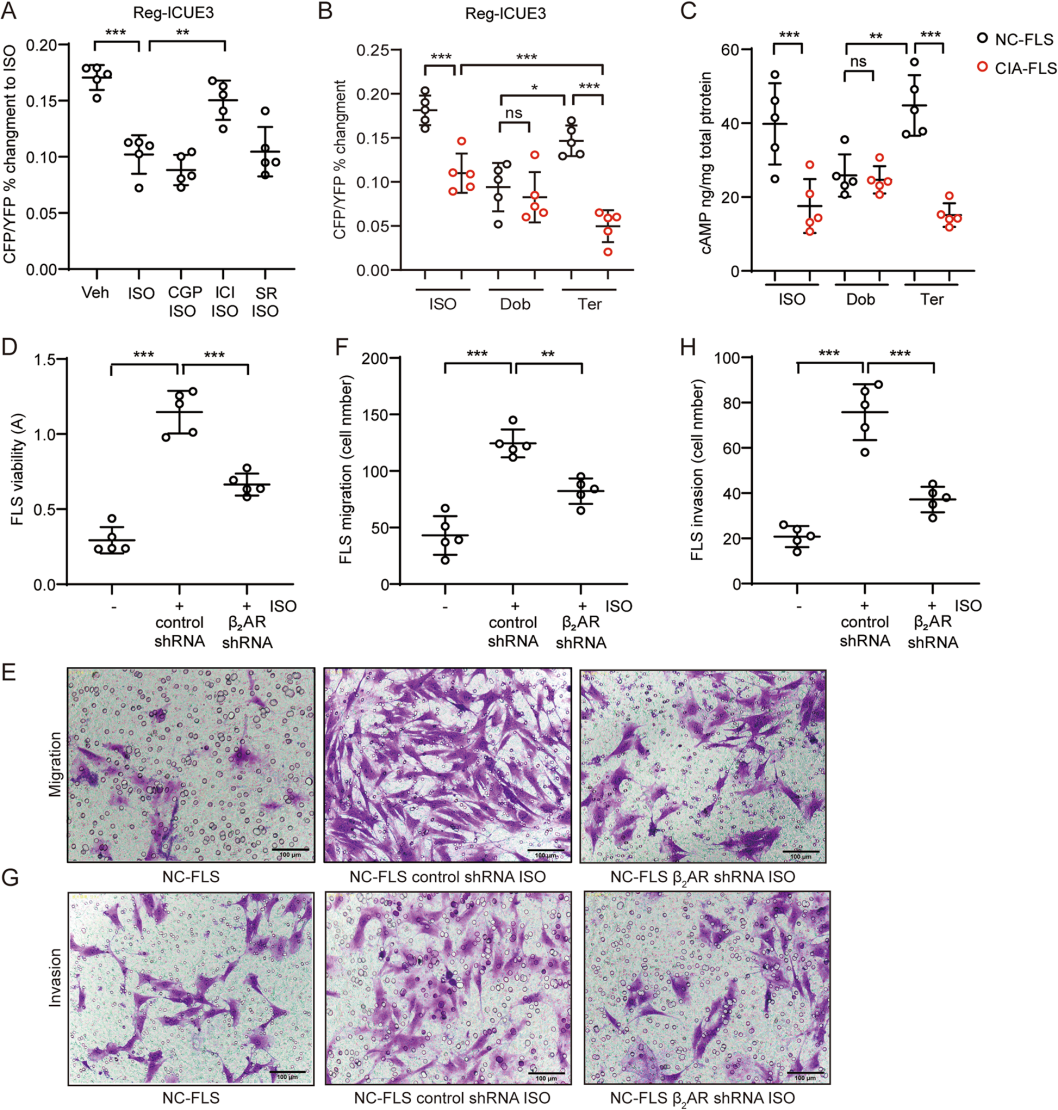
Chronic Epi stimulation inhibits cAMP production and activates FLSs through β_2_AR. **A** Intracellular cAMP production induced by ISO (100 nM) stimulation in normal rat FLSs that were treated with ISO (1 μM) in the presence or absence of CGP (1 μM), ICI (1 μM) or 100 nM SR overnight was detected in the FRET system. **B** Intracellular cAMP production in normal or CIA rat FLSs treated with Ter (10 μM), (10 μM), or ISO (1 μM) was detected in the FRET system. **C** The intracellular cAMP concentration in FLSs from normal or CIA rats treated with Ter or ISO was determined by a kit. **D** The effect of knocking down β_2_AR on FLS viability was detected by a CCK-8 assay. **E**, **F** The effects of knocking down β_2_AR on FLS migration were determined by a Transwell assay, and the data were analysed. Scale bar, 100 μm. **G**, **H** The effects of knocking down β_2_AR on FLS invasion were determined by a Transwell assay, and the data were analysed. Scale bar, 100 μm. The data are presented as the means ± SEMs; *p < 0.05, **p < 0.01, ***p < 0.001*; n* = 5 animals per group

To determine which isotype of βAR contributes to the reduced Epi response in FLSs under inflammatory conditions, we chronically stimulated normal rat FLSs with 1 μM ISO in the presence or absence of 1 μM CGP (β_1_AR antagonist), 1 μM ICI (β_2_AR antagonist), or 100 nM SR (β_3_AR antagonist) overnight and then evaluated the response to ISO using FRET. As measured, overnight ISO treatment significantly abated the cAMP synthesis response, which was restored by pretreatment with ICI (Fig. 2A), indicating that β_2_AR was functionally inhibited in FLSs under chronic overstimulation. Subsequently, we found that cAMP production in CIA FLSs was obviously inhibited in response to ISO or Ter challenge but was not inhibited in Dob-treated CIA FLSs (Fig. 2B), confirming that β_2_AR was dysfunctional in inflammatory FLSs. The FRET data were further verified with a cAMP detection kit, and identical results were obtained (Fig. 2C). As we found that β_2_AR was functionally impaired during chronic ISO stress, we knocked down β_2_AR using shRNA in normal rat FLSs and then subjected the cells to stimulation with 1 μM ISO to explore the role of β_2_AR dysfunction in FLS activation. Cells transfected with control shRNA was markedly activated by chronic ISO treatment, while ISO-induced proliferation of FLSs transfected with β_2_AR shRNA was obviously reduced (Fig. 2D). Similarly, migration and invasion were notably prevented in FLSs with β_2_AR knockdown compared with control cells after ISO treatment (Fig. 2E-H), suggesting that ISO-induced activation of FLSs is attributed to β2AR dysfunction.

### β_2_AR inhibits cAMP production in CIA-FLSs by coupling with G_αi_ instead of G_αs_

**Fig. 3:**
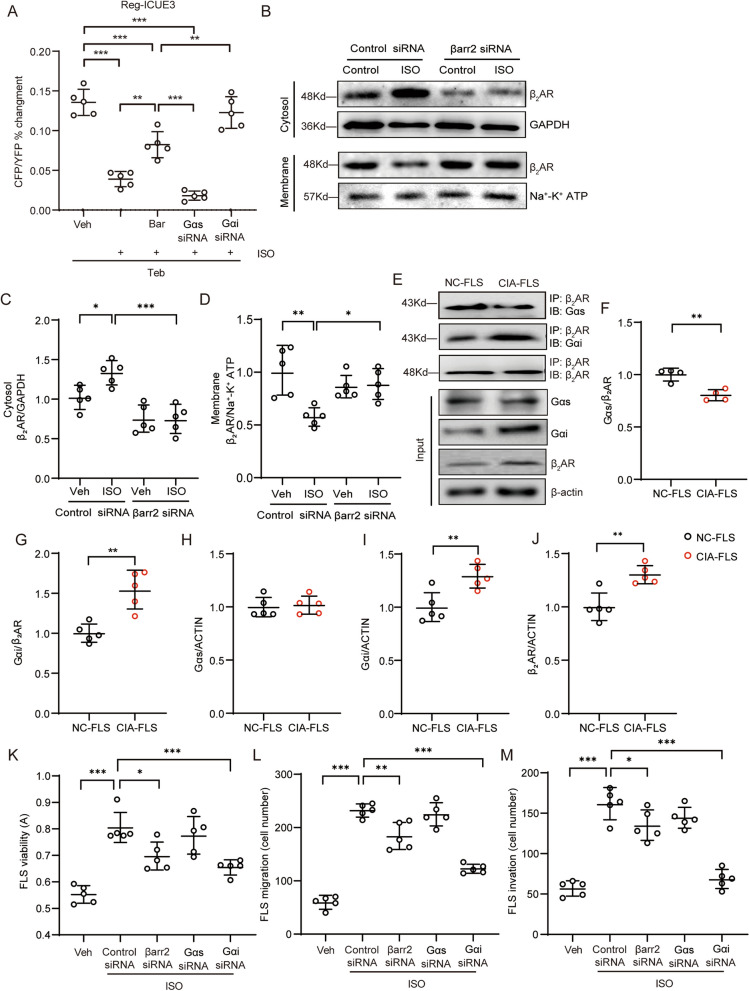
β_2_AR inhibits cAMP production in CIA-FLSs by coupling with G_αi_ instead of G_αs_. **A** Intracellular cAMP production in ISO (1 μM)-treated normal rat FLSs that were pretreated with Bar (10 μM), G_αs_ siRNA, or G_αi_ siRNA was detected in the FRET system. **B** The membrane and cytosolic distribution of β_2_AR after ISO stimulation was evaluated in normal and βarr2-deficient rat FLSs. **C** The cytosolic expression of β_2_AR was quantified. **D** The membrane expression of β_2_AR was quantified. **E–G** The binding of β_2_AR with G_αs_ or G_αi_ in FLSs from normal and CIA rats was determined by co-IP, and the data were analysed. **H-J** The expression of G_αs,_ G_αi_, and β_2_AR in normal and CIA rat FLSs was analysed using input samples. **K** The effect of knocking down βarr2, G_αs_, or G_αi_ on ISO-induced FLS viability was evaluated by a CCK-8 assay. **L** The effect of knocking down βarr2, G_αs_, or G_αi_ on ISO-induced FLS migration was analysed. **M** The effect of knocking down βarr2, G_αs_, or G_αi_ on ISO-induced FLS invasion was analysed. The data are presented as the means ± SEMs; *p < 0.05, *p < 0.05, **p < 0.01, ***p < 0.001*; n* = 4–5 animals per group

Basically, β_2_AR couples with G_αs_, resulting in the production of cAMP under stimulation, but high-level and long-term stimulation with ISO significantly attenuated cAMP production induced by the selective β_2_AR agonist Ter in FLSs. Bar partially recovers β_2_AR-cAMP signalling at the high catecholamine status. FLSs transfected with G_αs_ siRNA prior to ISO chronic stimulation could not response to Ter challenge. However, FLSs transfected with G_αi_ siRNA could produce abundant cAMP upon Ter stimulation even have been pretreated with high concentration of ISO (Fig. 3A). It is well known that β_2_AR can be desensitized and internalized via βarr2 under ligand stimulation. Indeed, we observed that ISO stimulation promoted the intracellular distribution of β_2_AR, while the abundance of membrane β_2_AR was decreased. Downregulation of βarr2 expression by siRNA clearly inhibited the internalization of β_2_AR in response to ISO stimulation (Fig. 3B-D), indicating that the internalization of β_2_AR was βarr2 dependent. In addition, preventing β_2_AR desensitization and internalization using Bar partially restored β_2_AR cAMP production under high catecholamine conditions, suggesting that βarr2 may partially contribute to the reduced function of β_2_AR during RA. Furthermore, we found that depletion of G_αs_ by a specific siRNA was not able to restore Ter-induced signalling, but surprisingly, inhibiting G_αi_ expression by siRNA transfection successfully restored Ter-induced cAMP production (Fig. 3A), indicating that high-level stimulation with ISO may lead to G_αi_ coupling of β_2_AR. The Co-IP assay revealed that in normal FLSs, β_2_AR was primarily bound by G_αs_ but exhibited limited binding of G_αi_; however, in CIA FLSs, more G_αi_ was coupled to β_2_AR, with decreased G_αs_ binding (Fig. 3E-G). In addition, in CIA FLSs, G_αs_ expression was not notably changed, but G_αi_ and β_2_AR expression was elevated (Fig. 3H-J). We further performed immunofluorescence staining to confirm the correlation between β_2_AR (green) and G_αs_/G_αi_ (red) in normal and CIA FLSs. The correlation ratio, Pearson correlation coefficient and overlap coefficient between β_2_AR and G_αs_ were decreased in CIA FLSs compared with normal cells (Supplementary Fig. 2A, C-E). In contrast, the correlation parameters between β_2_AR and G_αi_ were obviously increased in CIA FLSs (Supplementary Fig. 2B, F-H). In addition, we further explored the pathological downstream molecules that are responsible for acquisition of the CIA phenotype in normal FLSs under high-Epi conditions. G_αs_, G_αi_, and βarr2 were individually knocked down in rat FLSs by transfection of specific siRNAs, and the phenotype of FLSs in response to ISO stimulation was observed. Stimulating cells with ISO clearly promoted the proliferation, migration and invasion of normal rat FLSs. Deletion of βarr2 slightly inhibited ISO-induced FLS activation, and depletion of G_αs_ minimally prevented ISO-induced FLS overactivation, including the increases in proliferation, migration and invasion. However, knocking down G_αi_ effectively inhibited ISO-induced FLS hyperplasia and attenuated the arthritic morphology (Fig. 3K-M, Supplementary Fig. 3A and B). These results suggest that in addition to desensitization of β_2_AR, the coupling of β_2_AR to G_αi_ is the primary event responsible for the acquisition of the CIA phenotype by normal FLSs under β adrenergic stress; however, the underlying mechanism is unknown.

### The increased G_αi_ binding of β_2_AR is attributed to GRK2 and can be restored by a GRK2 inhibitor

**Fig. 4:**
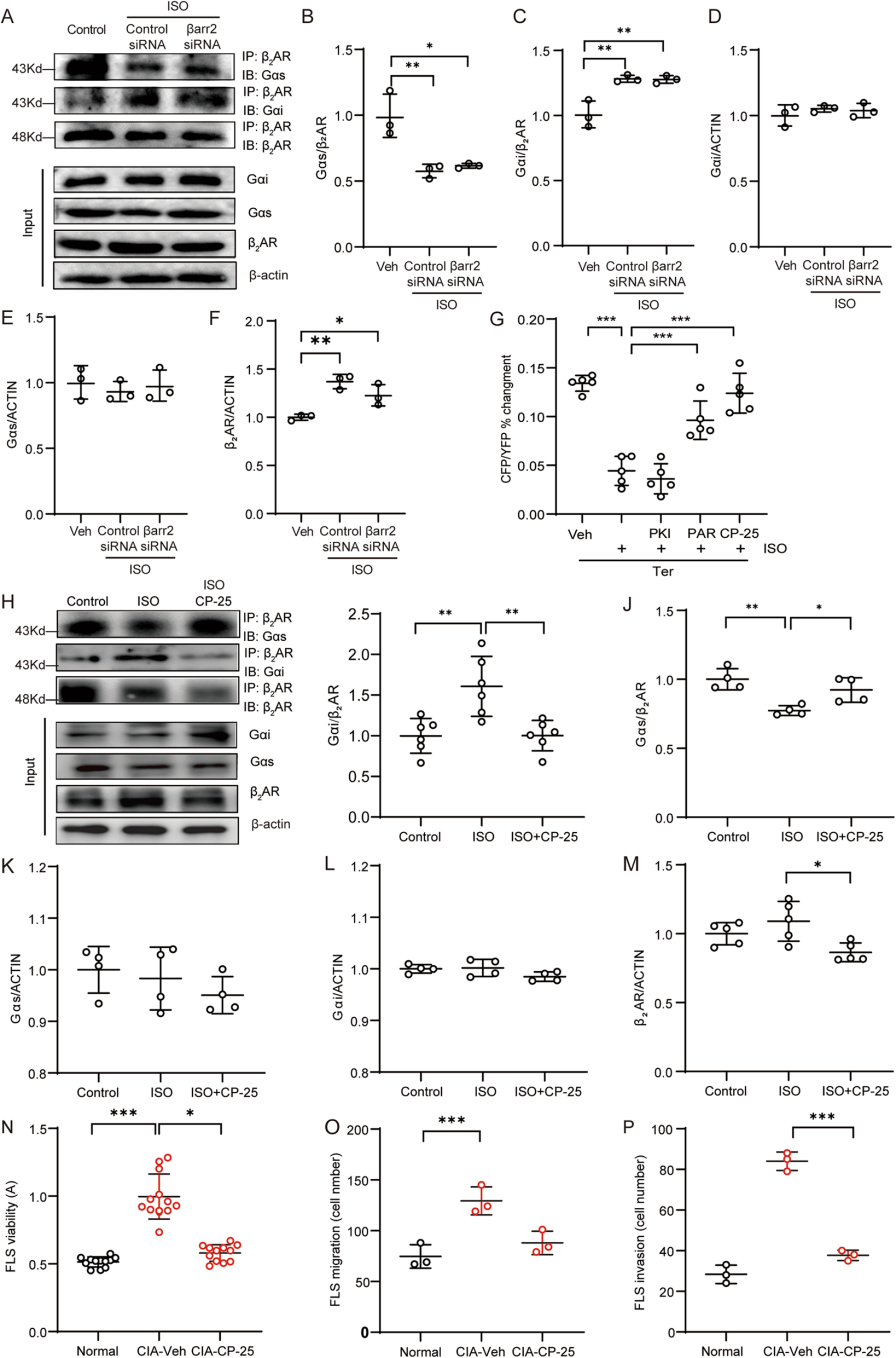
The increased G_αi_ coupling to β_2_AR is attributed to GRK2 and can be restored by a GRK2 inhibitor. **A-C** The correlation of β_2_AR with G_αs_ or G_αi_ in βarr2 knockdown FLSs after ISO stimulation was evaluated by co-IP. **D-F** The expression of G_αs,_ G_αi_, and β_2_AR in βarr2-depleted rat FLSs in response to ISO stimulation was analysed using input samples. **G** Intracellular cAMP production induced by Ter (10 μM) in ISO (1 μM)-treated normal rat FLSs that were pretreated with PKI (1 μM), PAR (1 μM), or CP-25 (1 μM) was detected in the FRET system. **H-J** The binding of β_2_AR with G_αs_ or G_αi_ in rat FLSs treated with ISO or ISO + CP-25 was evaluated by co-IP, and the data were analysed. The expression of (**K**) G_αs_, (**L**) G_αi_ or (**M**) β_2_AR in input samples of FLSs from the indicated treated rats was detected by Western blotting. (**N**) The viability of CIA FLSs treated with ISO or ISO + CP-25 was evaluated by a CCK-8 assay. **O** The numbers of invaded cells in the different groups were compared. **P** The numbers of migrated cells in the different groups were compared. The data are presented as the means ± SEMs; *p < 0.05, **p < 0.01, ***p < 0.001*; n* = 3–5 animals per group

As revealed, β_2_AR undergoes internalization upon chronic ISO stimulation through βarr2. We wanted to verify whether the ISO-induced coupling of G_αi_ to β_2_AR is also βarr2 dependent. However, the data showed that knocking down βarr2 minimally affected ISO-induced β_2_AR-G_αi_ coupling and that the binding of β_2_AR to G_αs_ in ISO-stimulated FLSs was not obviously changed when βarr2 was knocked down (Fig. 4A-F). These results confirmed that ISO-induced β_2_AR-G_αs_ coupling was not mediated by βarr2. Under physiological conditions, in response to ligand binding, β_2_AR is activated, resulting in the production of cAMP, which in turn results in β_2_AR phosphorylation by PKA. Moreover, the activation of β_2_AR results in the recruitment and activation of GRK2, which can also phosphorylate β_2_AR and regulate downstream signalling. The PKA inhibitor PKI, commercial GRK2 inhibitor PAR, and novel GRK2 inhibitor CP-25 were then used to pretreat normal rat FLSs prior to incubation with a high concentration of ISO overnight [15], and the FRET assay data showed that β_2_AR stimulation by Ter failed to induce cAMP production in either ISO- or PKI + ISO-treated FLSs, but either PAR or CP-25 restored the β_2_AR response (Fig. 4A), indicating that the desensitization of β_2_AR during Epi stress is mediated by GRK2 but not PKA. As expected, CP-25 specifically prevented the increased binding of G_αi_ to β_2_AR in ISO-treated rat FLSs and restored G_αs_ coupling (Fig. 4B-D). However, in vitro CP-25 treatment did not significantly change the expression of G_αs_ and G_αi_ induced by ISO but slightly inhibited the expression of β_2_AR, which was upregulated by ISO (Fig. 4B, E-G). The proliferation of FLSs from CIA rats was effectively inhibited by CP-25 treatment compared with Veh treatment in vitro (Fig. 4H). In addition, CP-25 successfully reduced the migration and invasion of CIA FLSs (Fig. 4I-K). These data indicate that high-level Epi stimulation-induced β_2_AR-G_αi_ coupling is dependent on GRK2 and that blocking this pathological change by treatment with a selective GRK2 inhibitor may effectively inhibit hyperplasia of CIA FLSs.

### CIA in rats is substantially ameliorated by treatment with a GRK2 inhibitor, accompanied by marked inhibition of FLS hyperplasia

**Fig. 5:**
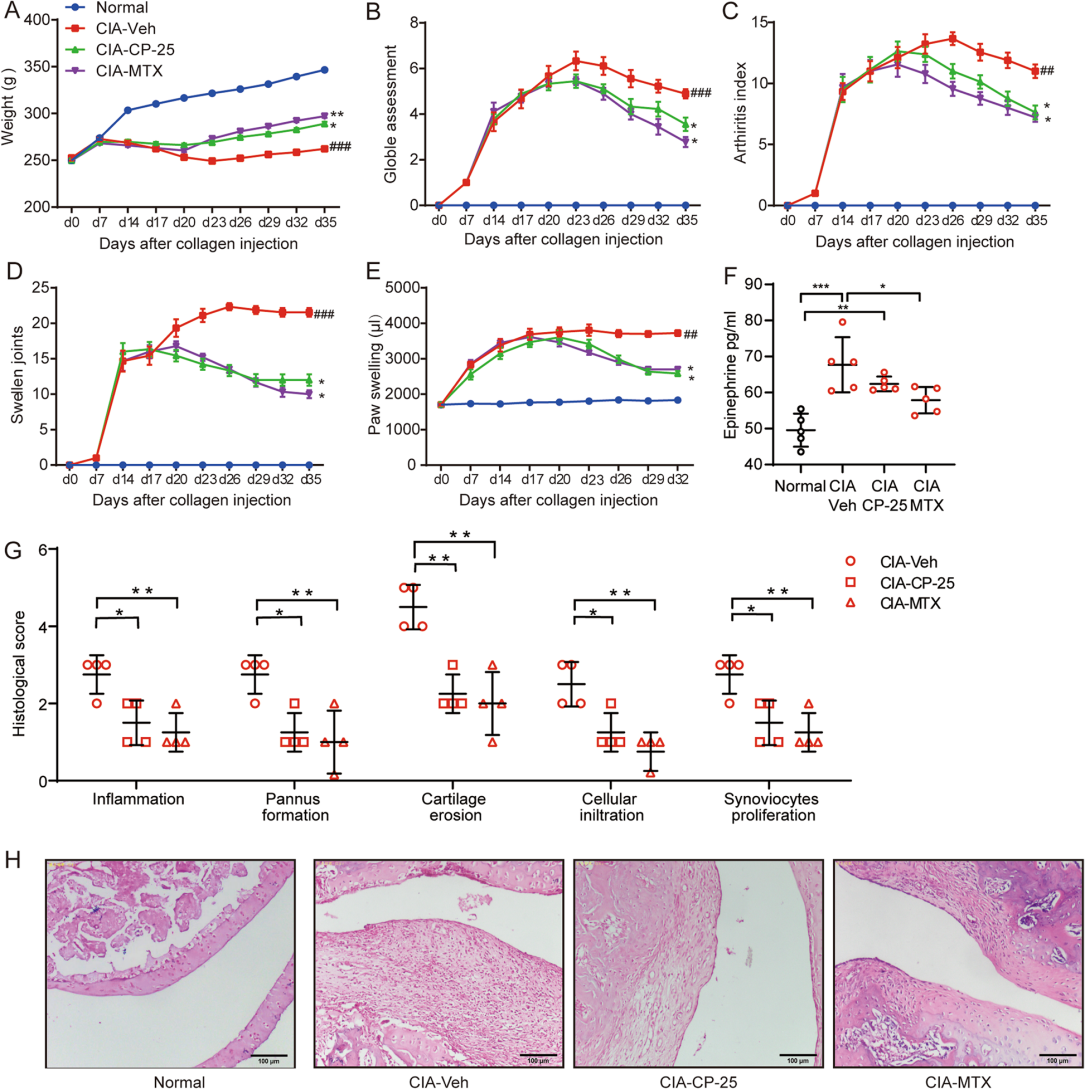
Rat CIA is substantially ameliorated by a GRK2 inhibitor, which markedly inhibits FLS hyperplasia. The (**A**) Body weight, **B** global assessment score, **C** arthritis index, **D** number of swollen joints, and **E** volume of the right hindpaw were recorded at the indicated time points. The data are presented as the means ± SEMs; ^*##*^*p* < 0.01, ^*###*^*p* < 0.001, CIA-Veh *vs.* Normal group; *p < 0.05, **p < 0.01, treated group *vs.* CIA-Veh group. **F** The serum concentration of Epi in treated rats was measured by ELISA. **G** Analysis of joint pathology in the different groups. **H** Representative images of synovial tissue pathology in the different groups. Scale bar, 100 μm. The data are presented as the means ± SEMs; *p < 0.05, **p < 0.01, ***p < 0.001*; n* = 5 animals per group

**Fig. 6:**
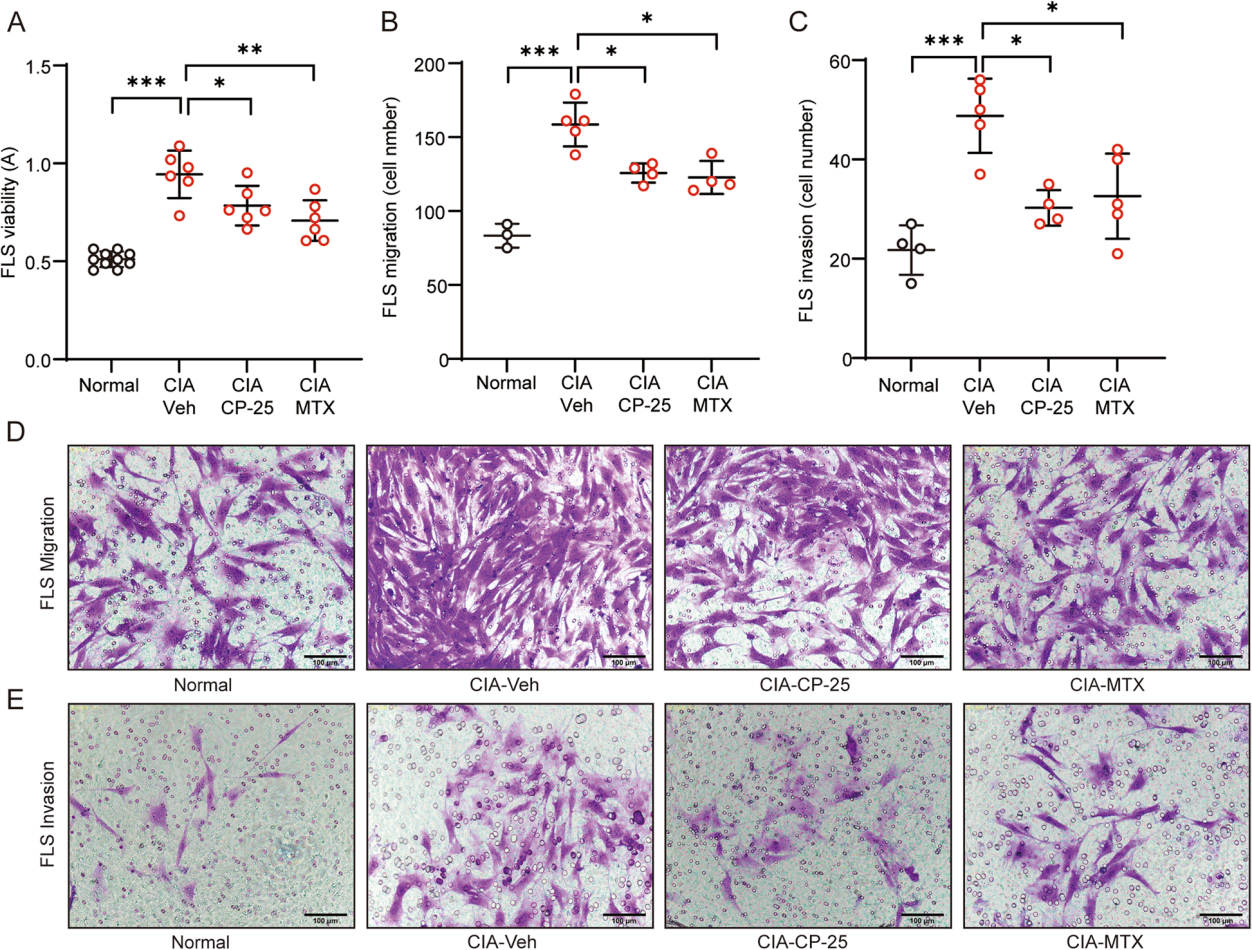
CP-25 treatment significantly prevents CIA FLS activation. **A** The viability of FLSs in different groups was evaluated by a CCK-8 assay. **B** The numbers of migrated cells in the different groups were compared. **C** The numbers of invaded cells in the different groups were compared. **D** The migration of FLSs in the different groups was evaluated by a Transwell assay. Scale bar, 100 μm. **E** The invasion of FLSs in different groups was evaluated by a Transwell assay. Scale bar, 100 μm. The data are presented as the means ± SEMs; *p < 0.05, **p < 0.01, ***p < 0.001*; n* = 3–5 animals per group

Considering the above results, we used CP-25 to treat CIA rats in vivo, with MTX as a positive control. The body weight of CIA rats was effectively restored by MTX administration (Fig. 5A). The increases in the global assessment score, arthritis index, number of swollen joints and paw swelling volume in CIA rats were substantially reduced by CP-25 or MTX treatment (Fig. 5B-E). However, CP-25 did not notably influence the increase in the serum Epi level in CIA rats; in contrast, MTX treatment significantly reduced Epi secretion (Fig. 5F), confirming that CP-25 restores the β_2_AR response by restoring receptor sensitivity per se but not by affecting the circulating Epi level. Joint histological analysis showed that both CP-25 and MTX were able to significantly reduce joint inflammation, synovial pannus formation, cartilage destruction, and immune cell infiltration, as well as synoviocyte proliferation (Fig. 5G and H). Then, FLSs were isolated from rats that received individual treatment, and cell function was analysed. As expected, in vivo administration of CP-25 or MTX effectively inhibited the proliferation, migration and invasion of CIA FLSs (Fig. 6A-E). Taken together, in this work, we observed upregulated expression of Epi in the joints of CIA rats, which led to the elevation of β_2_AR expression in CIA FLSs, accompanied by a switch in the coupling of G_αi_ in a GRK2-dependent manner, resulting in FLS hyperplasia and severe joint inflammation. Inhibition of GRK2 by CP-25 effectively prevented the G_αs_-G_αi_ switch, restored the response of β_2_AR in the setting of CIA and ultimately inhibited FLS activation and joint inflammation (Fig. 6F). These data reveal that the switch in G_αs_ to G_αi_ coupling to β_2_AR under adrenergic stress is an important pathomechanism of FLS hyperplasia in RA and is an effective pharmacological target of the GRK2 inhibitor CP-25 in the treatment of experimental RA.

## Discussion

Synovial tissue is located in the inner layer of the joint cavity. In normal joints, FLSs are regularly arranged in one to three layers; however, in RA, synovial tissue becomes the target of inflammation and the initiator of joint destruction through its extensive proliferation and migration, as well as the formation of panni that invade cartilage and bone [19]. The tumour-like pathological change in RA FLSs makes them similar to immortalized cancer cells. Commonly used antirheumatic drugs mainly target the overactivated immune response in immune cells, and hyperplasia of FLSs is almost completely ignored. Investigating the molecular mechanisms underlying the abnormal proliferation, migration, and invasion of FLS in RA is of great importance for controlling the onset and progression of RA [20].

Cell proliferation is usually controlled by intracellular cAMP, which is accepted as an important antiproliferative second messenger via its roles in inhibiting mitogen-activated protein kinase activity, increasing the expression of the cell cycle inhibitors p21cip1 and p27kip1, and reducing the expression of Cyclin D1 and D3 [21]. Evidence has also shown that cAMP-mediated cell growth inhibition depends on cAMP-mediated activation of Ras-association proximate 1 (Rap1), which is a small G protein that interacts with Raf-1, preventing Ras-induced ERK activation and finally inhibiting cell proliferation [22]. However, the roles of cAMP signalling in cell proliferation, differentiation, and migration are contradictory under certain conditions, including a low cell density and defective organ repair. In the setting of partial hepatectomy, the cAMP-dependent downstream kinase PKA phosphorylates cAMP response element binding protein (CREB) and triggers the transcription of cAMP responsive element modulator (CREM), leading to the proliferation of hepatocytes [23]. As previously described, in confluent cells, cAMP inhibits proliferation by phosphorylating Rap1 and subsequently prevents the activation of ERK. In contrast, in subconfluent cells, cAMP promotes the activation of ERK and contributes to proliferation [4]. Therefore, the role of cAMP in cell growth is dependent on the disease and extracellular microenvironment.

Many G_αs_-coupled GPCRs are expressed on FLSs, among which βARs are important receptors for the sympathetic neurotransmitter Epi, which has been found to be greatly enriched in the joint microenvironment. As reported, the sympathetic nervous system is activated by inflammation, and arthritis induced by CFA injection can facilitate neuroma formation by sympathetic nerve fibres [24], therefore, inflammation may contribute to the increased release of Epi in joints. Epi activates both αARs and βARs, and they have all been revealed to play pathological roles in the pathogenesis of inflammatory arthritis [25]. To clarify how the high-Epi environment in cells influences and regulates the activation of FLSs and the function of αARs and βARs, Epi was used to stimulate normal FLSs in vitro in combination with specific αAR and βAR antagonists. Moreover, specific αAR and βAR agonists were applied to confirm the pathological roles of each receptor. The findings were consistent with previous in vivo studies showing that treatment with neither nonselective nor selective αAR antagonists effectively ameliorated arthritis, but treatment with two β2AR antagonists was medicative [11]. Therefore, we demonstrated that a high level of Epi is able to promote FLS proliferation in vitro, accompanied by a reduced β adrenergic response, suggesting a pathological change in and effect of Epi-βAR signalling in FLS hyperplasia.

βARs normally couple with G_αs_ and promote cAMP production after activation. In contrast, β_3_AR has been reported to couple with G_αi_ but not G_αs_ in human cardiac myocytes and thus inhibit the activity of adenylyl cyclase and prevent cAMP production [26]. Regarding the three isotypes, β_1_AR is primarily located in the cardiovascular system, β_3_AR is mainly expressed by adipocytes, and β_2_AR is widely distributed and involved in the pathogenesis of many chronic diseases [27]. All three isotypes of receptors were detected in both normal and CIA FLSs, and the data revealed that the expression levels of β_1_AR and β_3_AR were not different between normal and CIA FLSs; in particular, β_3_AR was minimally expressed in FLSs, indicating that the inhibitory effect of β_3_AR on cAMP production in FLSs could be ignored. However, β_2_AR protein expression was significantly upregulated in CIA FLSs. However, commercial antibodies have been reported to lack specificity for βARs [28–32]. Therefore, we further measured the mRNA expression of all three βARs by qRT‒PCR using specific primers and found that the mRNA level of β_2_AR was elevated in inflammatory FLSs, consistent with the protein profile. To determine the βAR isotype that contributes to the impaired β adrenergic response, a receptor-selective agonist and antagonist were applied, pointing out the pathological effect of β_2_AR on cAMP production upon ISO stimulation in rheumatic FLSs. Knocking down β_2_AR effectively prevented ISO-induced FLS activation, migration and invasion, confirming that β_2_AR dysfunction leads to FLS activation under Epi stress.

However, β_2_AR has been revealed to have dual regulatory effects on inflammation, with inconclusive mechanisms. We observed upregulated expression of β_2_AR in CIA FLSs, but its cAMP induction ability was impaired. Studies have revealed a desensitization and internalization process of β_2_AR when it is overactivated in a GRK2- and βarr2-dependent manner. βArr2 acts as a scaffold protein to internalize GRK2-phosphorylated receptors through β_2_-adaptin-mediated clathrin-coated pits on the cell membrane [33]. We previously reported that in RA FLSs, the expression of both GRK2 and βarr2 is significantly upregulated [34]. Bar is a novel inhibitor of the βarr2-β_2_-adaptin interaction and specifically blocks β_2_AR internalization. Pretreatment with Bar only partially restored β_2_AR signalling under ISO stress, indicating that receptor internalization contributes to β_2_AR impairment, but there are some other mechanisms for β_2_AR dysfunction. Although it has been revealed that β_2_AR is able to couple with G_αi_ physiologically in a G_αs_-dominant manner [35], here, we demonstrated that a switch in G_αs_-G_αi_ coupling to β_2_AR is induced by ISO overstimulation and that this process is initiated by GRK2, since inhibiting GRK2 activity effectively prevented the switch. This result suggests that β_2_AR preferentially binds to G_αi_ during the process of inflammation and that this change may be due to the conformational change after GRK2 phosphorylation.

Activation or upregulation of GRK2 has been detected in many chronic diseases, including autoimmune diseases, cardiovascular diseases and metabolic diseases [33]. PAR is a selective serotonin reuptake inhibitor used to treat depression and has been identified as a GRK2 inhibitor. PAR therapy can effectively relieve arthritis in AA rats, but its effect on the nervous system limits its application as an anti-inflammatory agent [36]. Therefore, the development of GRK2 inhibitors is an important research area. CP-25 is a derivative of paeoniflorin, which is the key ingredient of the commercial antirheumatic drug total glucosides of paeony. We have shown that the novel GRK2 inhibitor CP-25, which blocks the kinase domain of GRK2, could effectively ameliorate experimental RA [13]. Furthermore, in this work, we demonstrated that CP-25 treatment successfully restored intracellular cAMP homeostasis in FLSs under catecholaminergic stress by preventing GRK2-mediated predominant coupling of G_αi_ to β_2_AR.

**Fig. 7:**
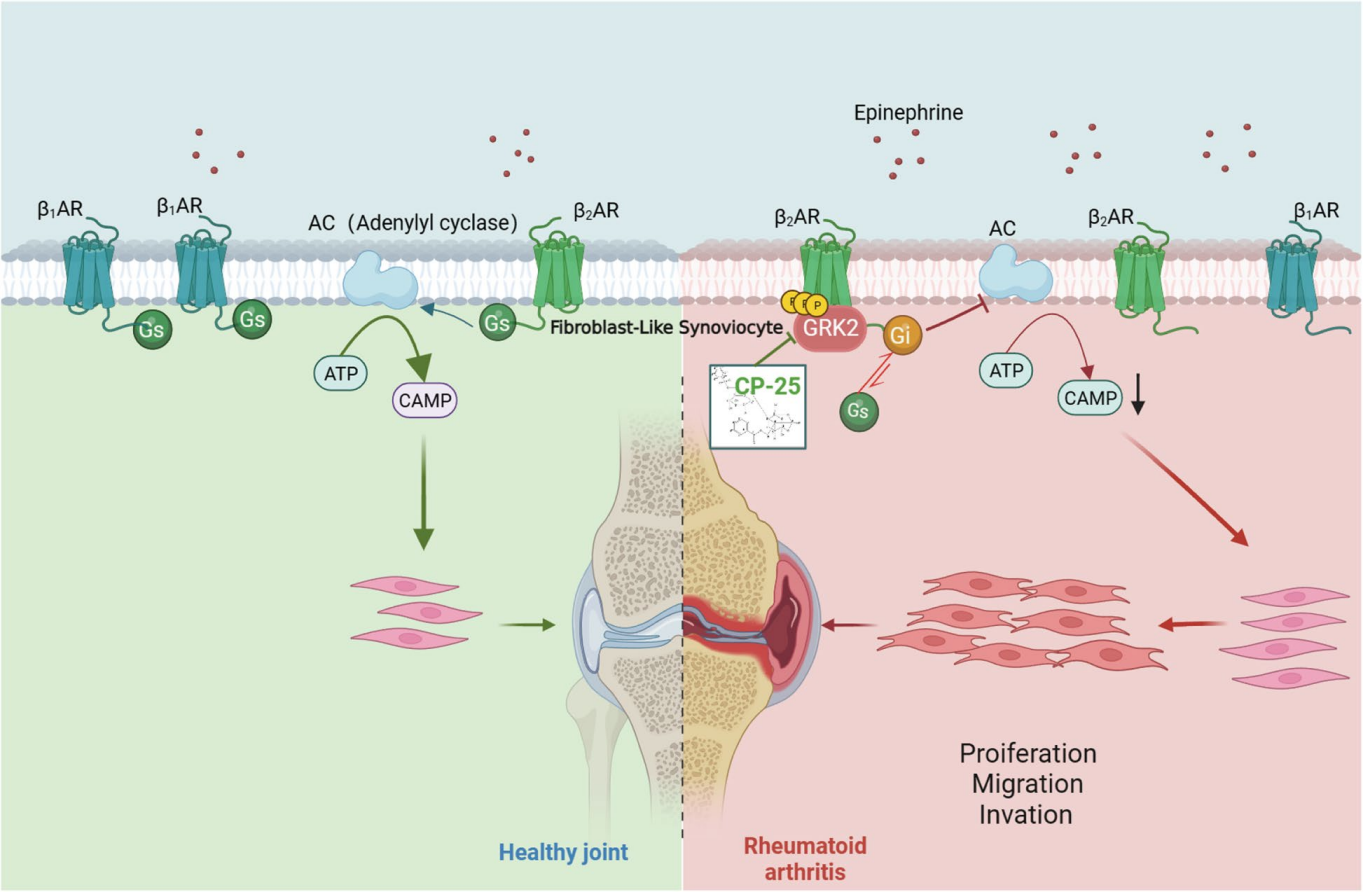
Graphical abstract. In normal synovial tissue, β-adrenergic receptors on FLSs activate adenylyl cyclase mainly by coupling with G_αs_, thereby maintaining a physiological intracellular cAMP level. In the inflammatory environment, increased Epi leads to a GRK2-mediated switch in G_αs_-G_αi_ coupling to β_2_AR on FLSs and a decrease in intracellular cAMP production and subsequently promotes FLS proliferation, migration and invasion, resulting in RA. The novel GRK2 inhibitor CP-25 inhibits the hyperactivation of rheumatic synoviocytes and alleviates CIA through restoration of G_αs_ coupling to β_2_AR and maintenance of the β_2_AR response in FLSs

In conclusion, we revealed that the catecholamine-enriched microenvironment in arthritic joints leads to a GRK2-mediated switch in G_αs_-G_αi_ coupling to β_2_AR on FLSs and to a decrease in intracellular cAMP production and finally promotes FLS hyperplasia, migration and invasion. The novel GRK2 inhibitor CP-25 inhibits the hyperactivation of rheumatic synoviocytes by restoring G_αs_ coupling to β_2_AR and maintaining the β_2_AR response in FLSs (Fig. 7).

### Supplementary Information

**Additional file 1.** Uncropped, original Western blot data using antibodies specific for β_1_AR, β_2_AR, G_αs_, G_αi_ and β-actin; corresponding to Fig. 1, Fig. 3, and Fig. 4.

**Additional file 2: Supplementary Fig. 1.** Arthritis manifestations in the joints of CIA rats. The (A) Body weight, (B) global assessment score, (C) arthritis index, (D) number of swollen joints, and (E) volume of the right hindpaw were recorded at the indicated time points. The data are presented as the means ± SEMs; ^*###*^*p*<0.001, CIA-Veh *vs.* Normal group; *n*=5 animals per group.

**Additional file 3: Supplementary Fig. 2.** The correlation between β_2_AR and G_αs_ or G_αs_ in normal and CIA FLSs was evaluated using immunofluorescence images. (A) The colocalization of β2AR and Gαs in both groups of rat FLSs was detected by immunofluorescence staining. Scale bar, 200 μm. (B) The colocalization of β2AR and Gαi in both groups of rat FLSs was detected by immunofluorescence staining. Scale bar, 200 μm. The data are presented as the means ± SEMs. (C) The correlation ratio, (D) Pearson correlation coefficient, and (E) overlap coefficient between β2AR and G_αs_ were analysed. (F) The correlation ratio, (G) Pearson correlation coefficient, and (H) overlap coefficient between β_2_AR and G_αi_ were analysed. The data are presented as the means ± SEMs; **p<0.01*.*

**Additional file 4: Supplementary Fig. 3.** The effect of βarr2, G_αs_, and G_αi_ on ISO-induced FLS migration and invasion. (A) The migration of ISO-induced normal rat FLSs with βarr2, G_αs_, or G_αi_ knockdown was evaluated by a Transwell assay. Scale bar, 100 μm. (B) The invasion of ISO-induced normal rat FLSs with βarr2, G_αs_, or G_αi_ knockdown was evaluated by a Transwell assay. Scale bar, 100 μm.

**Additional file 5: Supplementary Fig. 4.** The effect of CP-25 on the migration and invasion of CIA FLSs. The migration and invasion of CIA FLSs treated with ISO or ISO+CP-25 were evaluated by Transwell assays. Scale bar, 100 μm.

## Data Availability

All data generated or analysed during this study are included in this published article [and its supplementary information files].

## Abbreviations

AA: Adjuvant arthritis
ANOVA: Analysis of variance
Bar: Barbadin
βarr2: β-Arrestin2
β_2_AR: β_2_ Adrenergic receptor
cAMP: Cyclic 3′,5′-adenosine monophosphate
CCK-8: Cell counting kit-8
CFP: Cyan fluorescent protein
CIA: Collagen-induced arthritis
Co-IP: Coimmunoprecipitation
CP-25: Paeoniflorin-6′-O-benzene sulfonate
DMEM: Dulbecco’s Modified Eagle Medium
Dob: Dobutamine
Dex: Dexmedetomidine
ELISA: Enzyme-linked immunosorbent assay
Epi: Epinephrine
ERK: Extracellular regulated protein kinase
FLSs: Fibroblast-like synovial cells
FRET: Fluorescence resonance energy transfer
GPCRs: G protein-coupled receptors
GRK2: G protein coupled receptor kinase 2
HRP: Horseradish peroxidase
IL-1β: Interleukin-1β
ISO: Isoproterenol
MMPs: Matrix metalloproteinases
MTX: Methotrexate
PAR: Paroxetine
Phent: Phentolamine
Pheny: Phenylephrine
Prop: Propranolol
PKA: Protein kinase A
PKI: PKA inhibitor
RA: Rheumatoid arthritis
qRT‒PCR: Quantitative real-time PCR
SD: Standard deviation
SDS: Sodium dodecyl sulfate
shRNA: Short hairpin RNA
siRNA: Small interfering RNA
TBST: Tris-buffered saline containing 0.05% Tween 20
Ter: Terbutaline
TNF-α: Tumour necrosis factor-α
Veh: Vehicle
YFP: Yellow fluorescent protein
