# Supplementary material for: CP-25 inhibits the hyperactivation of rheumatic synoviocytes by suppressing the switch in Gαs-Gαi coupling to the β2-adrenergic receptor
Source: Cell Commun Signal. 2023 Nov 30;21:346. doi: 10.1186/s12964-023-01358-z (PMC10688045; doi:10.1186/s12964-023-01358-z)
Supplement: Supplementary file 2 — Additional file 1. Uncropped, original Western blot data using antibodies specific for β1AR, β2AR, Gαs, Gαi and β-actin; corresponding to Fig. 1, Fig. 3, and Fig. 4. [file 12964_2023_1358_MOESM1_ESM.pptx]

## Slide 1
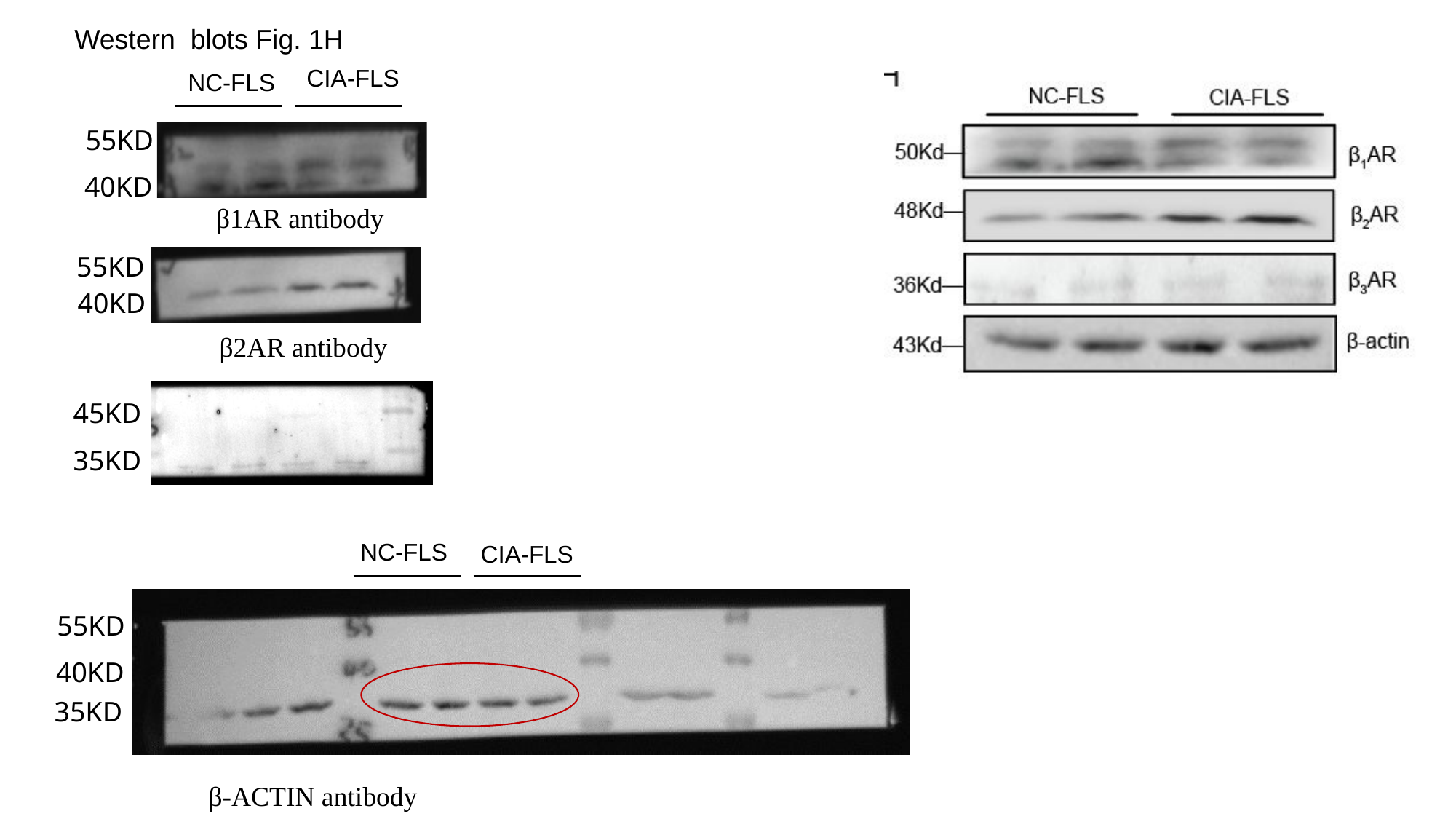

Western blots Fig. 1H
CIA-FLS
NC-FLS
55KD
40KD
β1AR antibody
55KD
40KD
β2AR antibody
45KD
35KD
NC-FLS
CIA-FLS
55KD
40KD
35KD
β-ACTIN antibody

## Slide 2
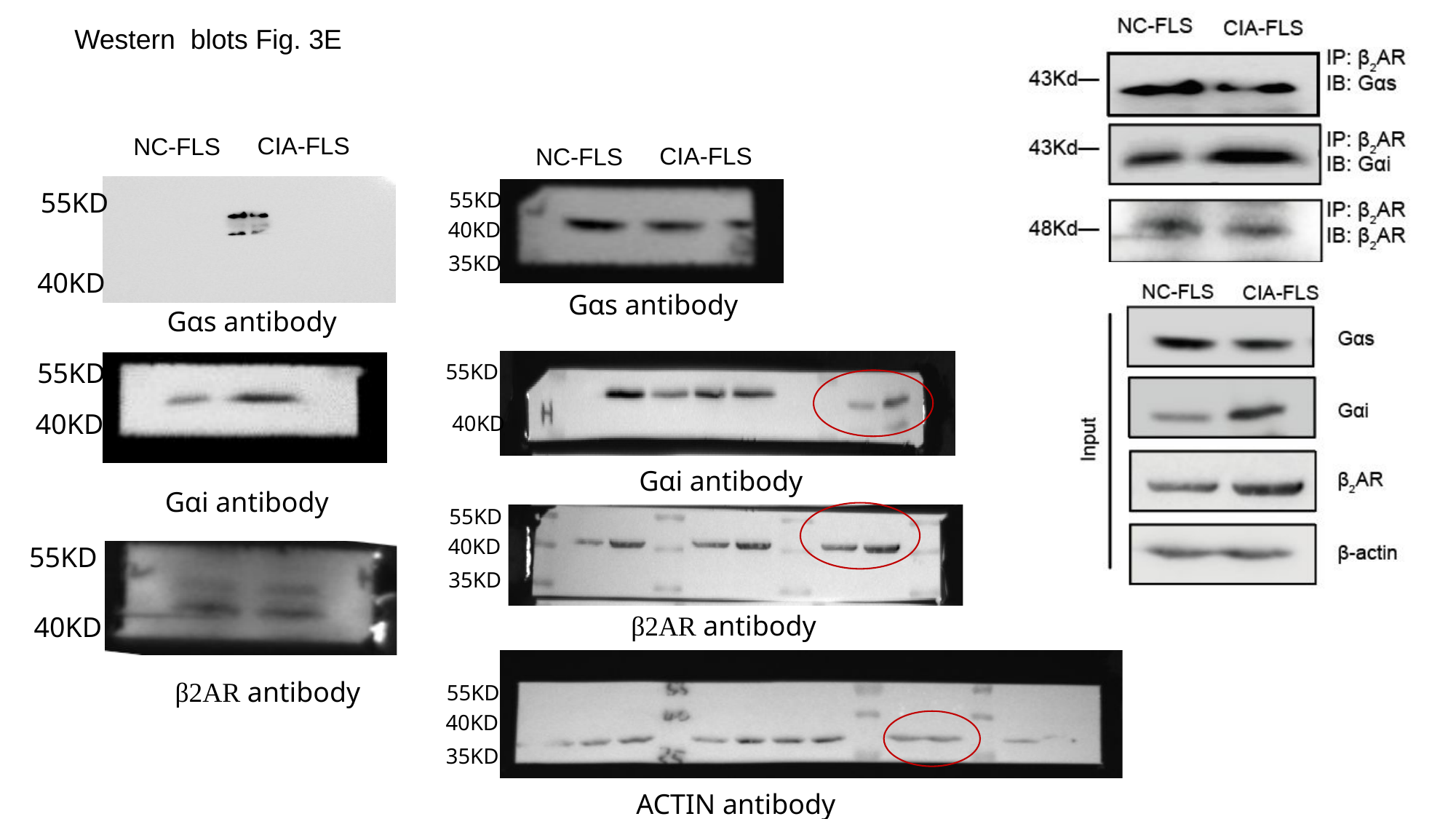

Western blots Fig. 3E
CIA-FLS
NC-FLS
CIA-FLS
NC-FLS
55KD
55KD
40KD
35KD
40KD
Gαs antibody
Gαs antibody
55KD
55KD
40KD
40KD
Gαi antibody
Gαi antibody
55KD
40KD
55KD
35KD
β2AR antibody
40KD
β2AR antibody
55KD
40KD
35KD
ACTIN antibody

## Slide 3
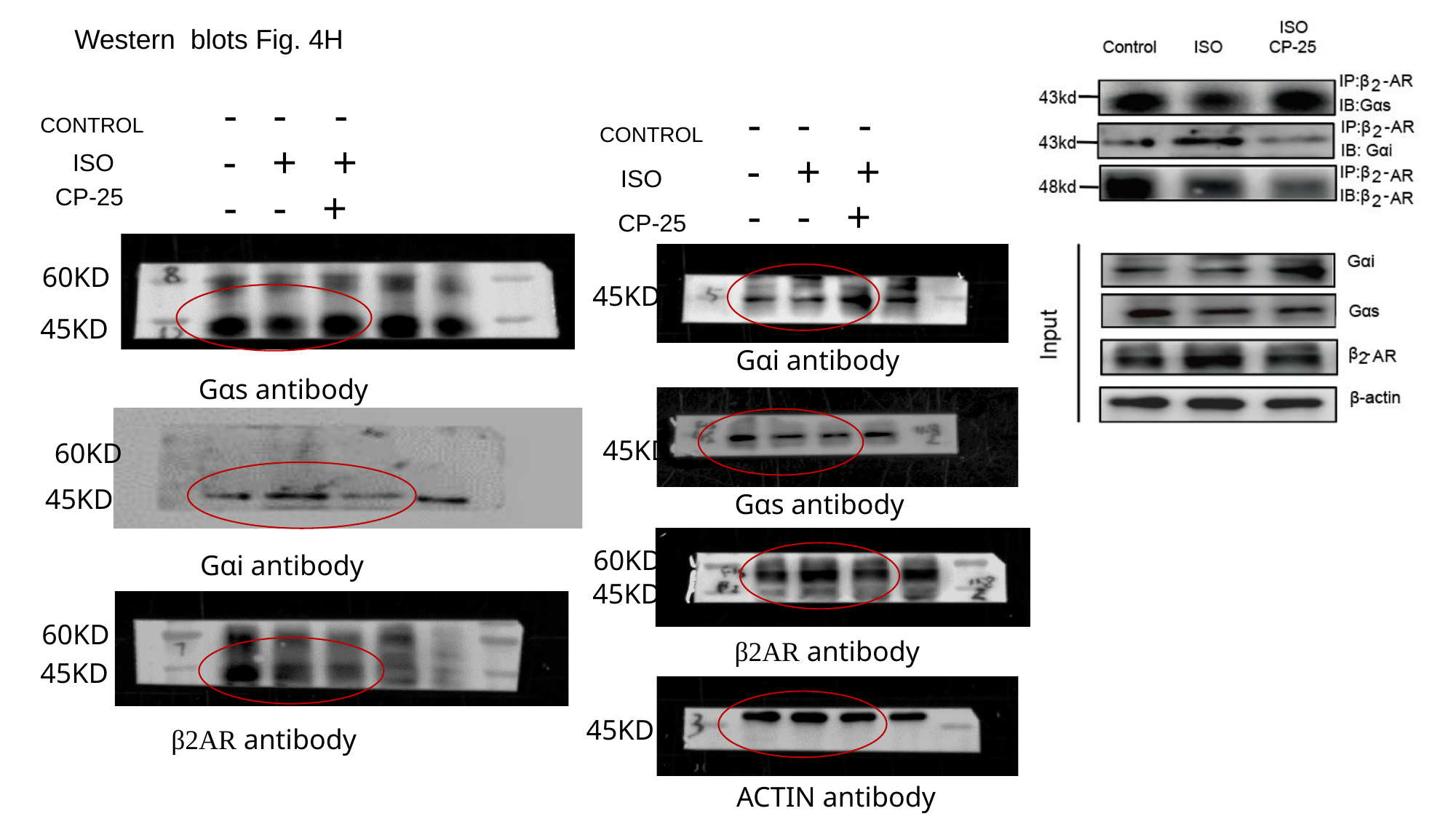

Western blots Fig. 4H
- - -
- - -
CONTROL
CONTROL
- + +
- + +
ISO
ISO
- - +
CP-25
- - +
CP-25
60KD
45KD
45KD
Gαi antibody
Gαs antibody
45KD
60KD
45KD
Gαs antibody
60KD
Gαi antibody
45KD
60KD
β2AR antibody
45KD
45KD
β2AR antibody
ACTIN antibody

## Slide 4
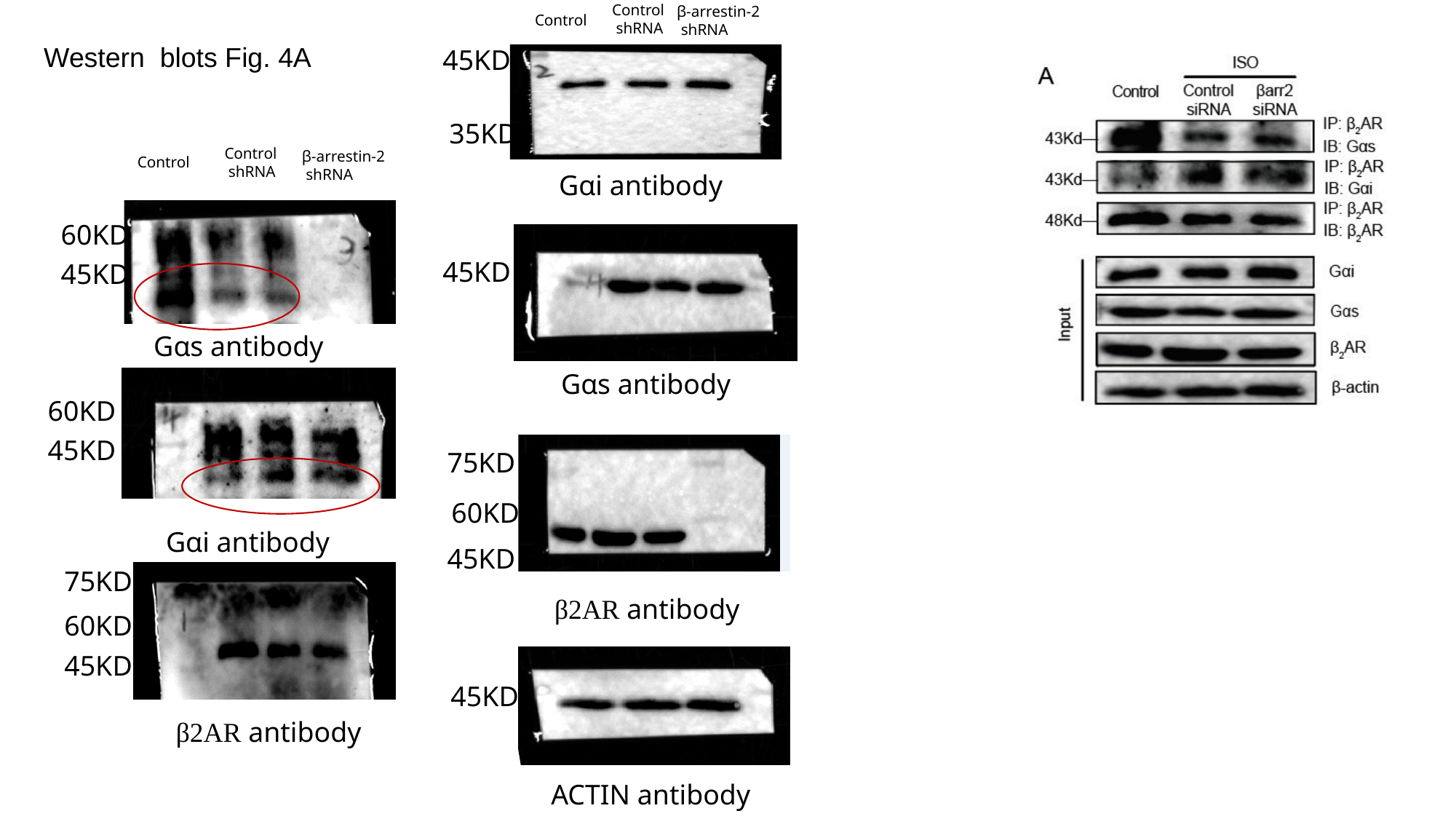

Control
 shRNA
β-arrestin-2
 shRNA
Control
Western blots Fig. 4A
45KD
35KD
Control
 shRNA
β-arrestin-2
 shRNA
Control
Gαi antibody
60KD
45KD
45KD
Gαs antibody
Gαs antibody
60KD
45KD
75KD
60KD
Gαi antibody
45KD
75KD
β2AR antibody
60KD
45KD
45KD
β2AR antibody
ACTIN antibody

## Slide 5
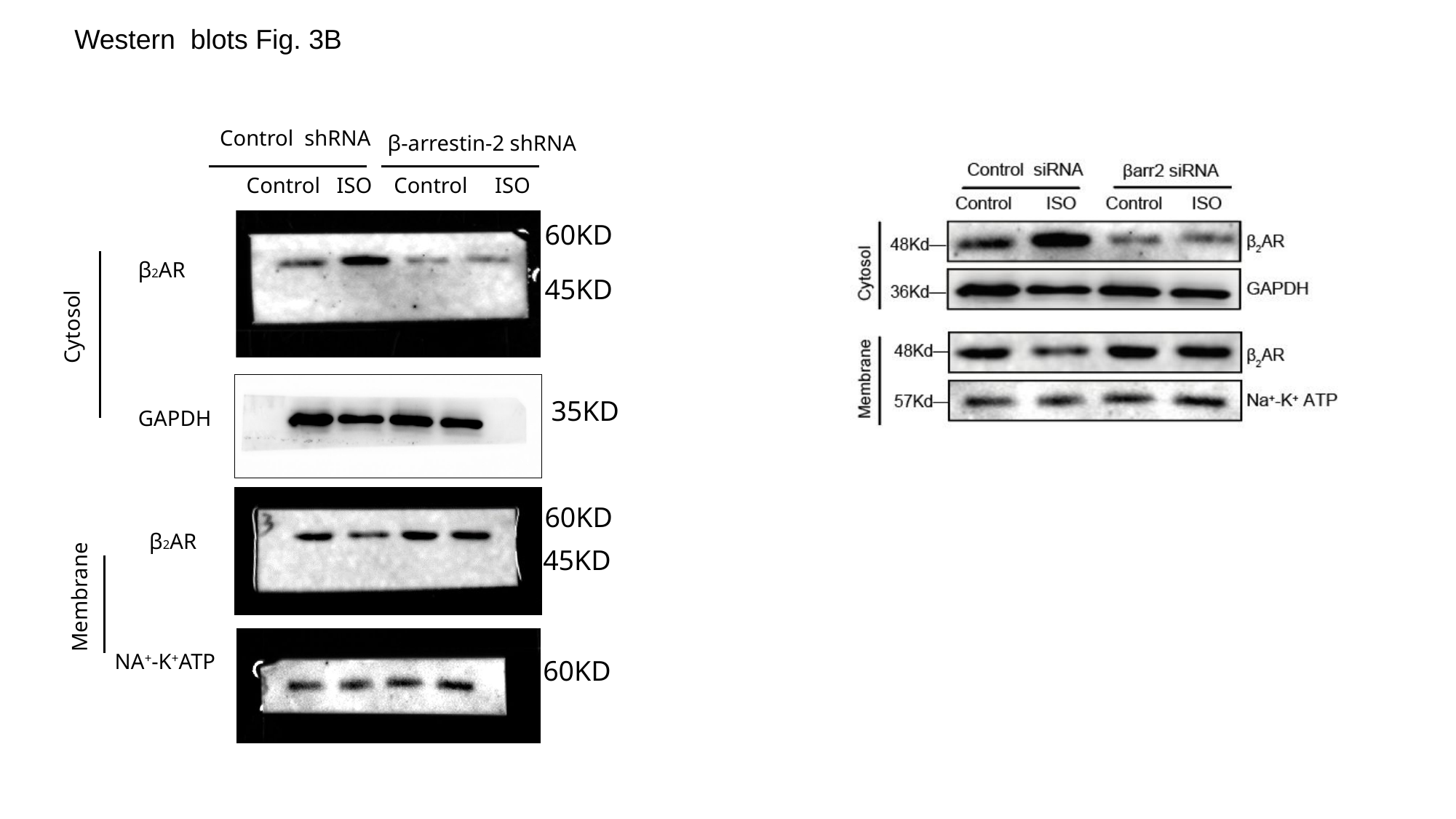

Western blots Fig. 3B
Control shRNA
β-arrestin-2 shRNA
Control ISO Control ISO
60KD
Cytosol
β2AR
45KD
35KD
GAPDH
60KD
β2AR
Membrane
45KD
NA+-K+ATP
60KD
