# Supplementary figures and images for: CP-25 inhibits the hyperactivation of rheumatic synoviocytes by suppressing the switch in Gαs-Gαi coupling to the β2-adrenergic receptor
Source: Cell Commun Signal. 2023 Nov 30;21:346. doi: 10.1186/s12964-023-01358-z (PMC10688045; doi:10.1186/s12964-023-01358-z)

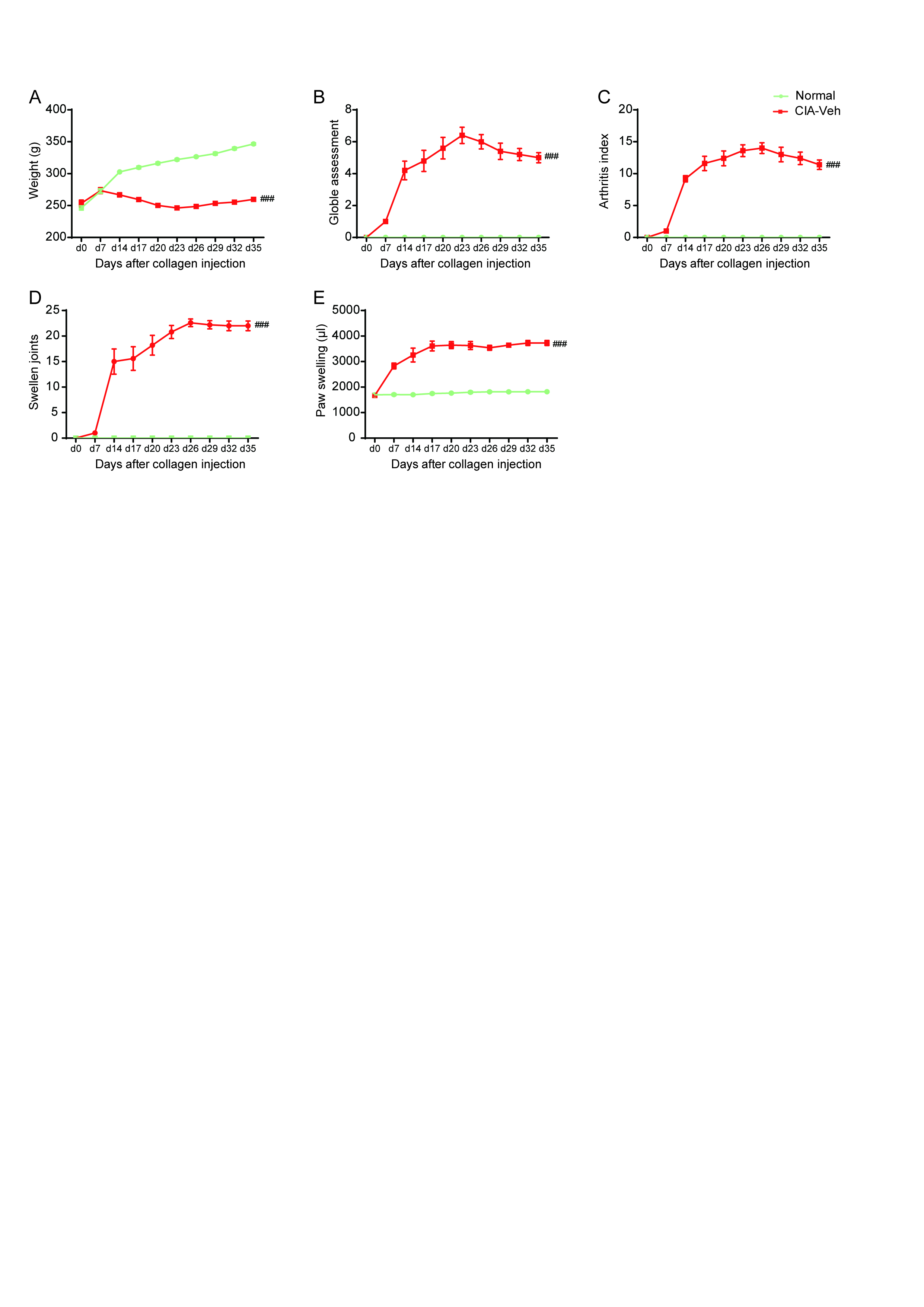

Supplement: Supplementary file 3 — Additional file 2: Supplementary Fig. 1. Arthritis manifestations in the joints of CIA rats. The (A) Body weight, (B) global assessment score, (C) arthritis index, (D) number of swollen joints, and (E) volume of the right hindpaw were recorded at the indicated time points. The data are presented as the means ± SEMs; ###p<0.001, CIA-Veh vs. Normal group; n=5 animals per group. [file 12964_2023_1358_MOESM2_ESM.tif]

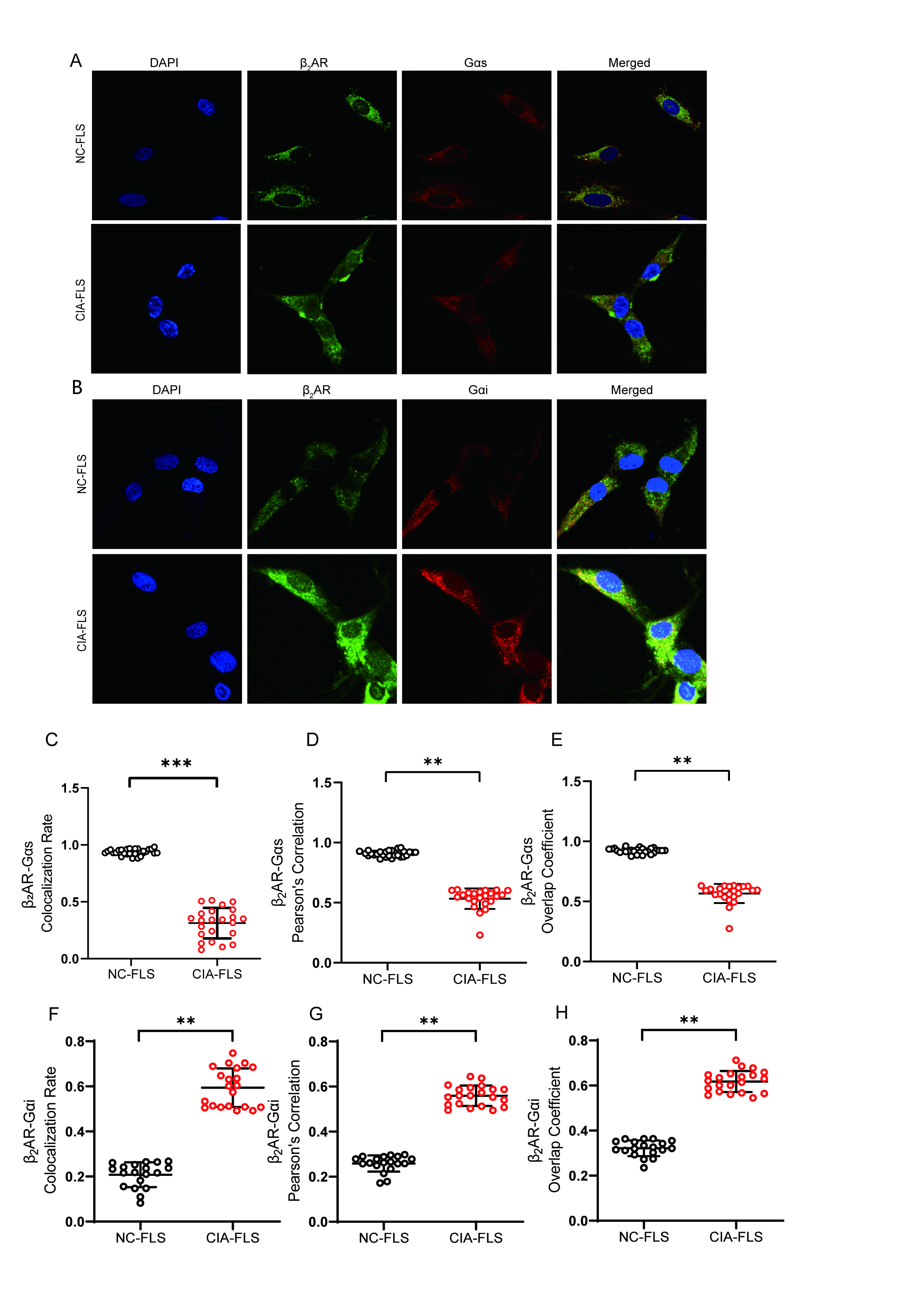

Supplement: Supplementary file 4 — Additional file 3: Supplementary Fig. 2. The correlation between β2AR and Gαs or Gαs in normal and CIA FLSs was evaluated using immunofluorescence images. (A) The colocalization of β2AR and Gαs in both groups of rat FLSs was detected by immunofluorescence staining. Scale bar, 200 μm. (B) The colocalization of β2AR and Gαi in both groups of rat FLSs was detected by immunofluorescence staining. Scale bar, 200 μm. The data are presented as the means ± SEMs. (C) The correlation ratio, (D) Pearson correlation coefficient, and (E) overlap coefficient between β2AR and Gαs were analysed. (F) The correlation ratio, (G) Pearson correlation coefficient, and (H) overlap coefficient between β2AR and Gαi were analysed. The data are presented as the means ± SEMs; **p<0.01. [file 12964_2023_1358_MOESM3_ESM.tif]

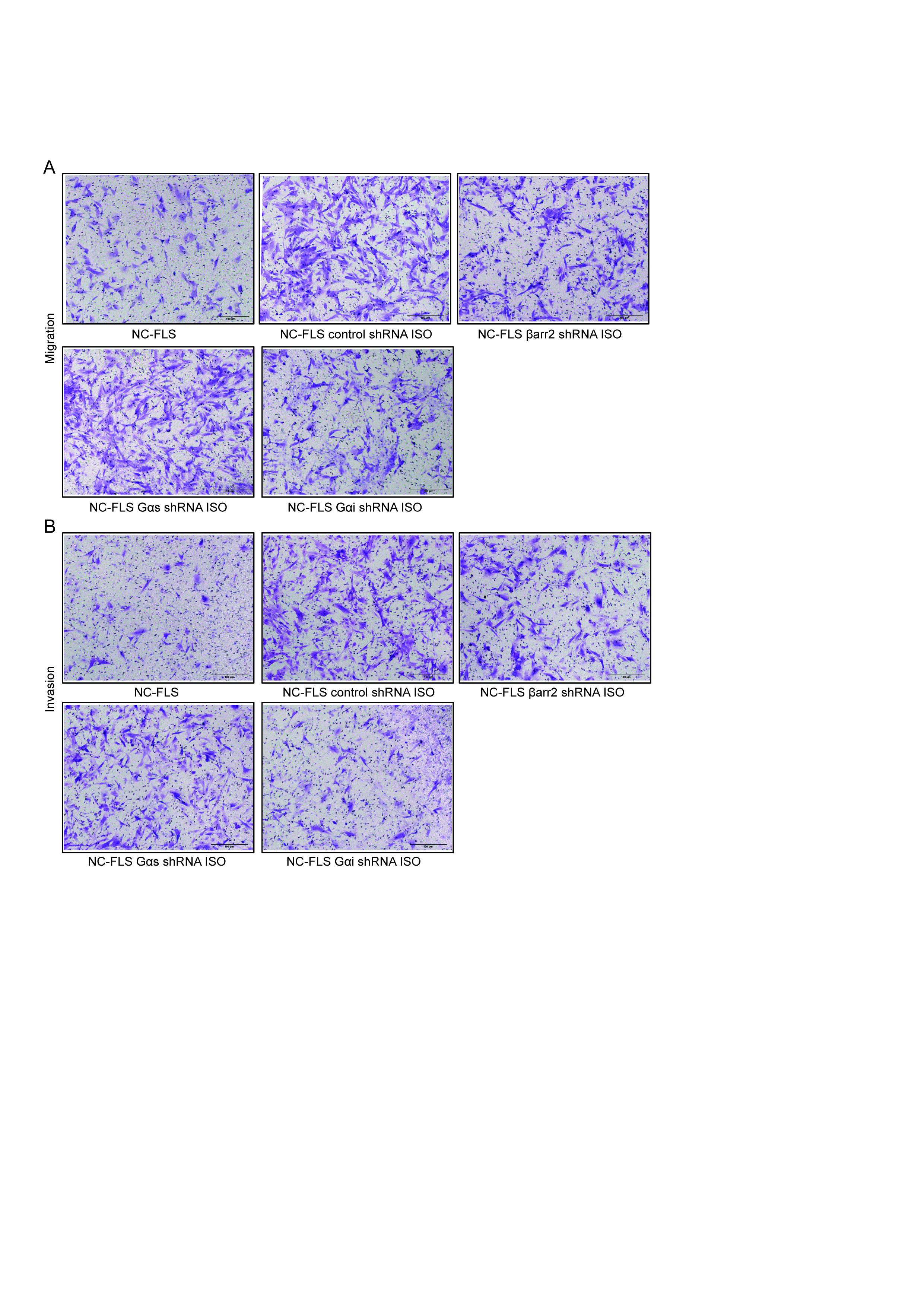

Supplement: Supplementary file 5 — Additional file 4: Supplementary Fig. 3. The effect of βarr2, Gαs, and Gαi on ISO-induced FLS migration and invasion. (A) The migration of ISO-induced normal rat FLSs with βarr2, Gαs, or Gαi knockdown was evaluated by a Transwell assay. Scale bar, 100 μm. (B) The invasion of ISO-induced normal rat FLSs with βarr2, Gαs, or Gαi knockdown was evaluated by a Transwell assay. Scale bar, 100 μm. [file 12964_2023_1358_MOESM4_ESM.tif]

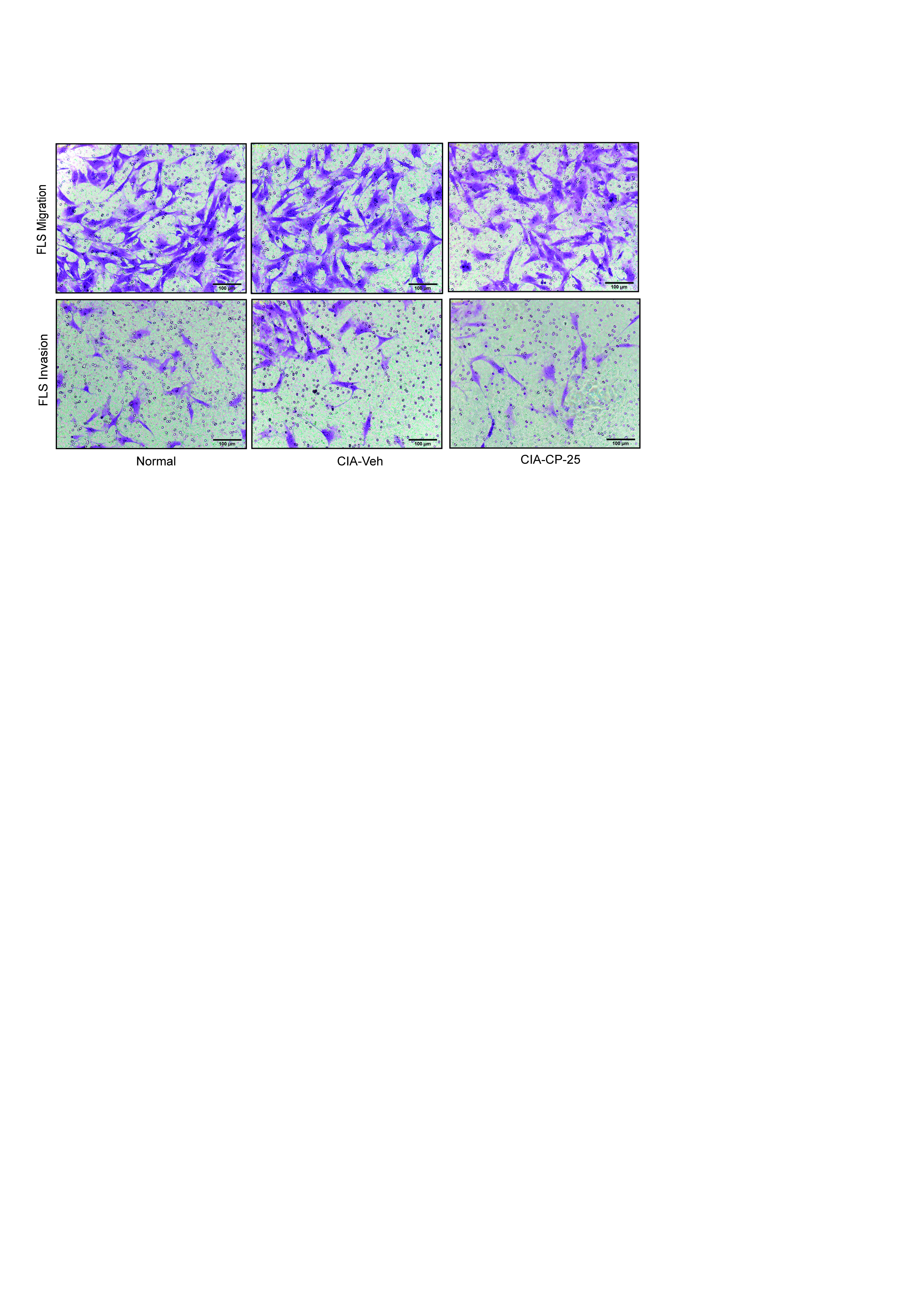

Supplement: Supplementary file 6 — Additional file 5: Supplementary Fig. 4. The effect of CP-25 on the migration and invasion of CIA FLSs. The migration and invasion of CIA FLSs treated with ISO or ISO+CP-25 were evaluated by Transwell assays. Scale bar, 100 μm. [file 12964_2023_1358_MOESM5_ESM.tif]
